# Correction: Mineralized belemnoid cephalic cartilage from the late Triassic Polzberg *Konservat-Lagerstätte* (Austria)

**DOI:** 10.1371/journal.pone.0315418

**Published:** 2024-12-05

**Authors:** Petra Lukeneder, Alexander Lukeneder

The Data Availability statement for this paper is incorrect. The correct statement is: All raw data and measurements are available in the Supporting Data File. 3D surface data will be made available upon publication in the https://zenodo.org/ data base (https://doi.org/10.5281/zenodo.6393725) and on the website of the Polzberg Project (https://www.nhm-wien.ac.at/forschung/geologie__palaeontologie/forschungsprojekte/polzberg).
