# Supplementary material for: Mineralized belemnoid cephalic cartilage from the late Triassic Polzberg Konservat-Lagerstätte (Austria)
Source: PLoS One. 2022 Apr 20;17(4):e0264595. doi: 10.1371/journal.pone.0264595 (PMC9020720; doi:10.1371/journal.pone.0264595)
Supplement: S1 Fig — (PDF) [file pone.0264595.s001.pdf]

Supporting Figure S3. SEM-EDS report for sample NHMW 2012/0117/0024 (Carbon).

C:\ESEM Daten\leon\Lukeneder\20210930\20210930_Knorpel_006.spc

Label:Knorpel 5

kV:20.0 Tilt:0.0 Take-off:34.7 Det:Octane Elect Plus

Res:128 Amp.T:3.84 FS:4048 Lsec:100 30-Sep-2021 11:45:28

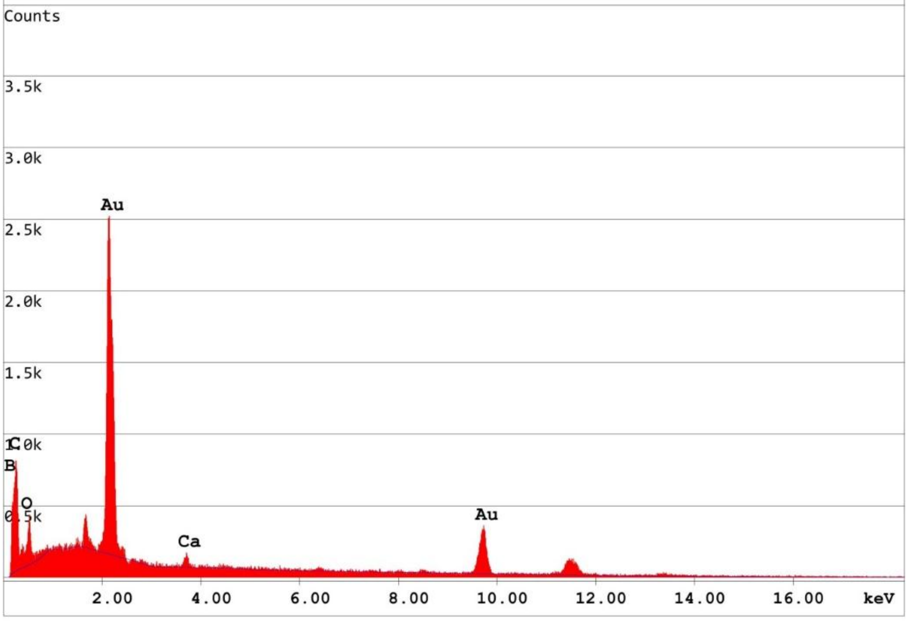

EDAX ZAF Quantification (Standardless)
Element Normalized
SEC Table : Default

Element	Wt %	At %	K-Ratio	Z	A	F
B K	12.20	26.88	0.0585	1.0785	0.4442	1.0002
C K	29.54	58.60	0.0610	1.1534	0.1790	1.0000
O K	5.24	7.81	0.0099	1.1335	0.1657	1.0000
CaK	0.60	0.36	0.0048	1.0651	0.7472	1.0000
AuL	52.42	6.34	0.4030	0.7519	1.0224	1.0000
Total	100.00	100.00				

Element	Net Inte.	Bkgd Inte.	Inte. Error	P/B
B K	20.33	1.25	2.35	16.26
C K	39.57	2.88	1.70	13.74
O K	16.88	5.29	3.10	3.19
CaK	7.65	7.81	6.31	0.98
AuL	43.81	4.38	1.65	10.00
