# Supplementary material for: Mineralized belemnoid cephalic cartilage from the late Triassic Polzberg Konservat-Lagerstätte (Austria)
Source: PLoS One. 2022 Apr 20;17(4):e0264595. doi: 10.1371/journal.pone.0264595 (PMC9020720; doi:10.1371/journal.pone.0264595)
Supplement: S2 Fig — (PDF) [file pone.0264595.s002.pdf]

Supporting Figure S4. SEM-EDS report for sample NHMW 2012/0117/0024 (calcitic fillings).

C:\ESEM Daten\leon\Lukeneder\20210930\20210930_Knorpel_005.spc

Label:Knorpel 5

kV:20.0 Tilt:0.0 Take-off:34.7 Det:Octane Elect Plus

Res:128 Amp.T:3.84 FS:4827 Lsec:100 30-Sep-2021 11:41:39

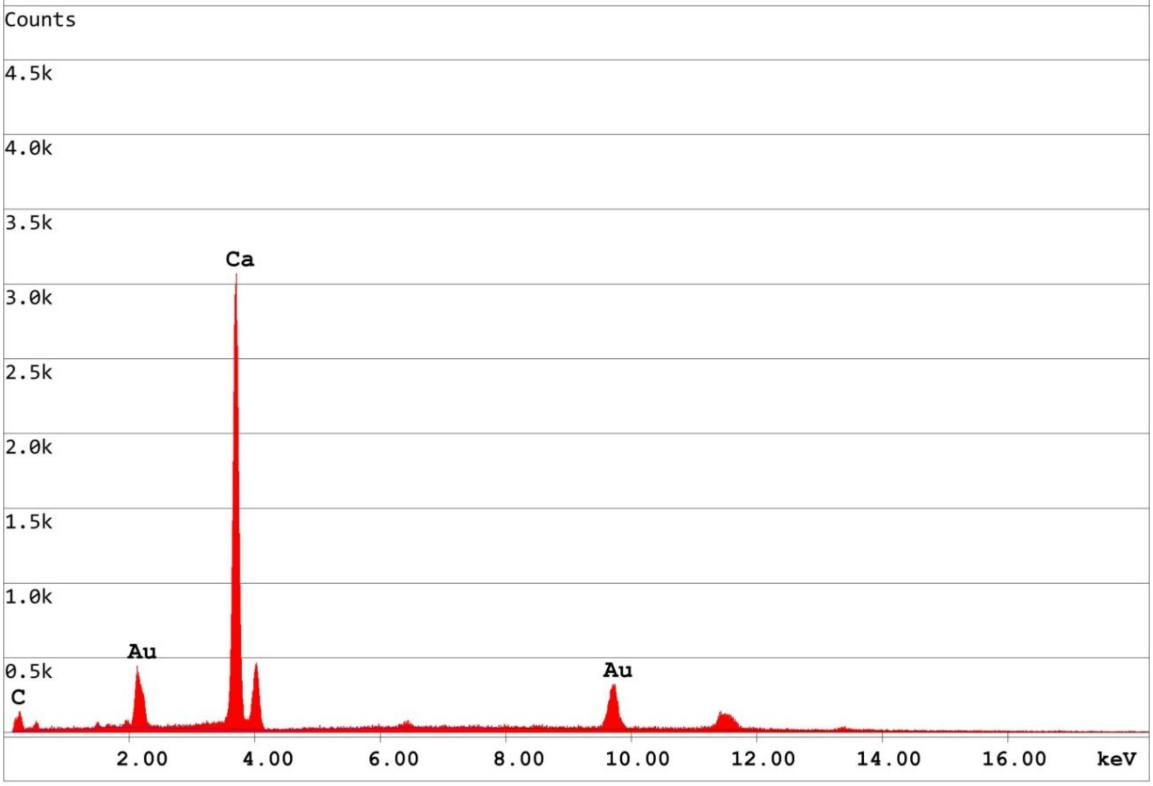

EDAX ZAF Quantification (Standardless)
Element Normalized
SEC Table : Default

Element	Wt %	At %	K-Ratio	Z	A	F
C K	5.51	29.91	0.0142	1.2408	0.2074	1.0004
CaK	29.96	48.73	0.2375	1.1520	0.6882	1.0000
AuL	64.53	21.36	0.5418	0.8332	1.0077	1.0000
Total	100.00	100.00				

Element	Net Inte.	Bkgd Inte.	Inte. Error	P/B
C K	6.34	0.43	4.23	14.74
CaK	260.75	2.34	0.62	111.43
AuL	40.58	3.74	1.71	10.85
