# Supplementary figures and images for: Mineralized belemnoid cephalic cartilage from the late Triassic Polzberg Konservat-Lagerstätte (Austria)
Source: PLoS One. 2022 Apr 20;17(4):e0264595. doi: 10.1371/journal.pone.0264595 (PMC9020720; doi:10.1371/journal.pone.0264595)

**Supporting Figure S7. Raman spectrum for specimen NHMW2021/0016/0397 measured at low-energy conditions.**

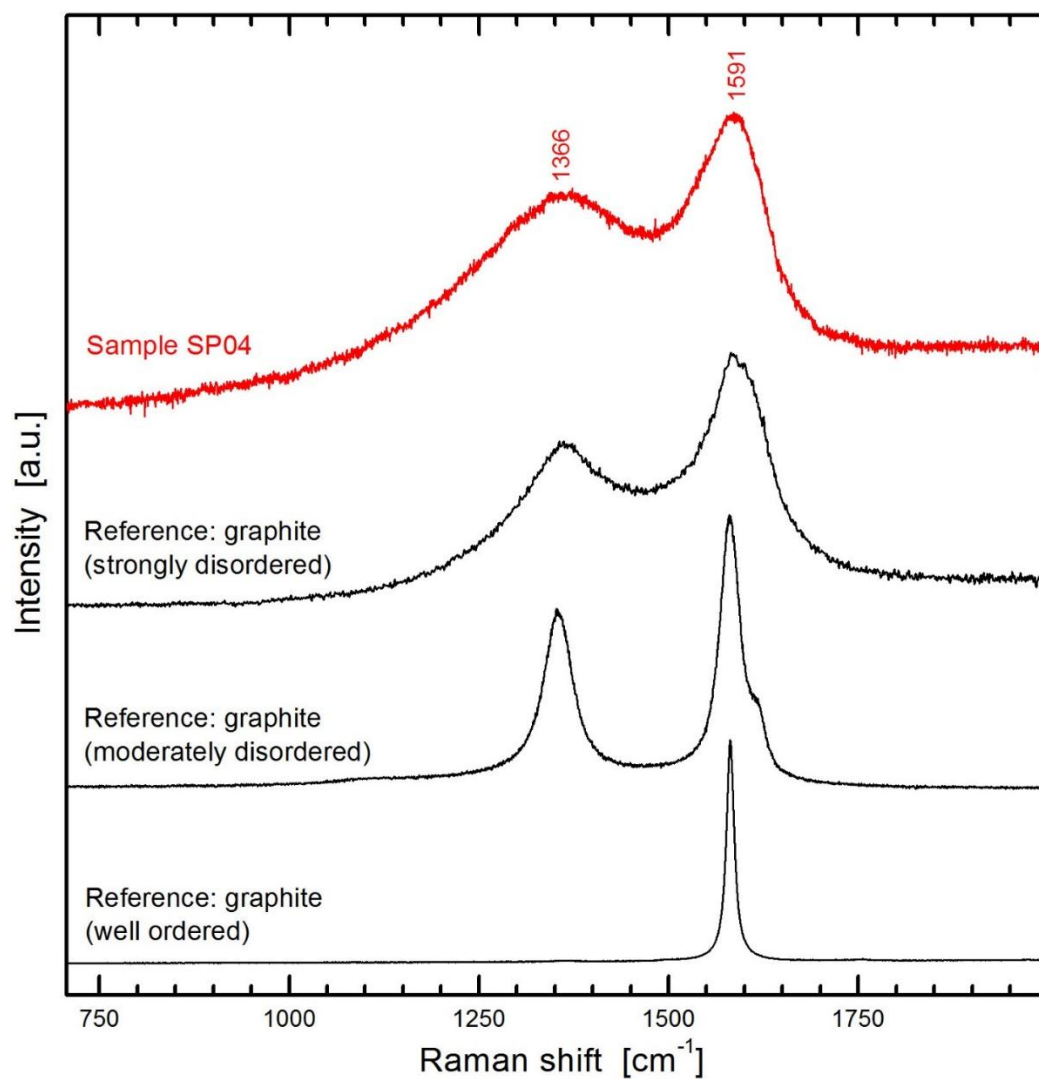

Supplement: S3 Fig — (PDF) [file pone.0264595.s003.pdf]
