# Supplementary material for: Mineralized belemnoid cephalic cartilage from the late Triassic Polzberg Konservat-Lagerstätte (Austria)
Source: PLoS One. 2022 Apr 20;17(4):e0264595. doi: 10.1371/journal.pone.0264595 (PMC9020720; doi:10.1371/journal.pone.0264595)

Supporting Figure S8. Cephalic cartilage of Loligo vulgaris. A Specimen of *Loligo vulgaris*, red arrows marking position of cephalic cartilage. **B** Well developed cephalic cartilage of *Loligo vulgaris* specimen from cranial view, yellow arrow pointing to opening for esophagus. **C** Cephalic cartilage from lateral view, exhibiting ocular cartilage. cc cephalic cartilage; oc ocular cartilage. Scale bars 1 cm.

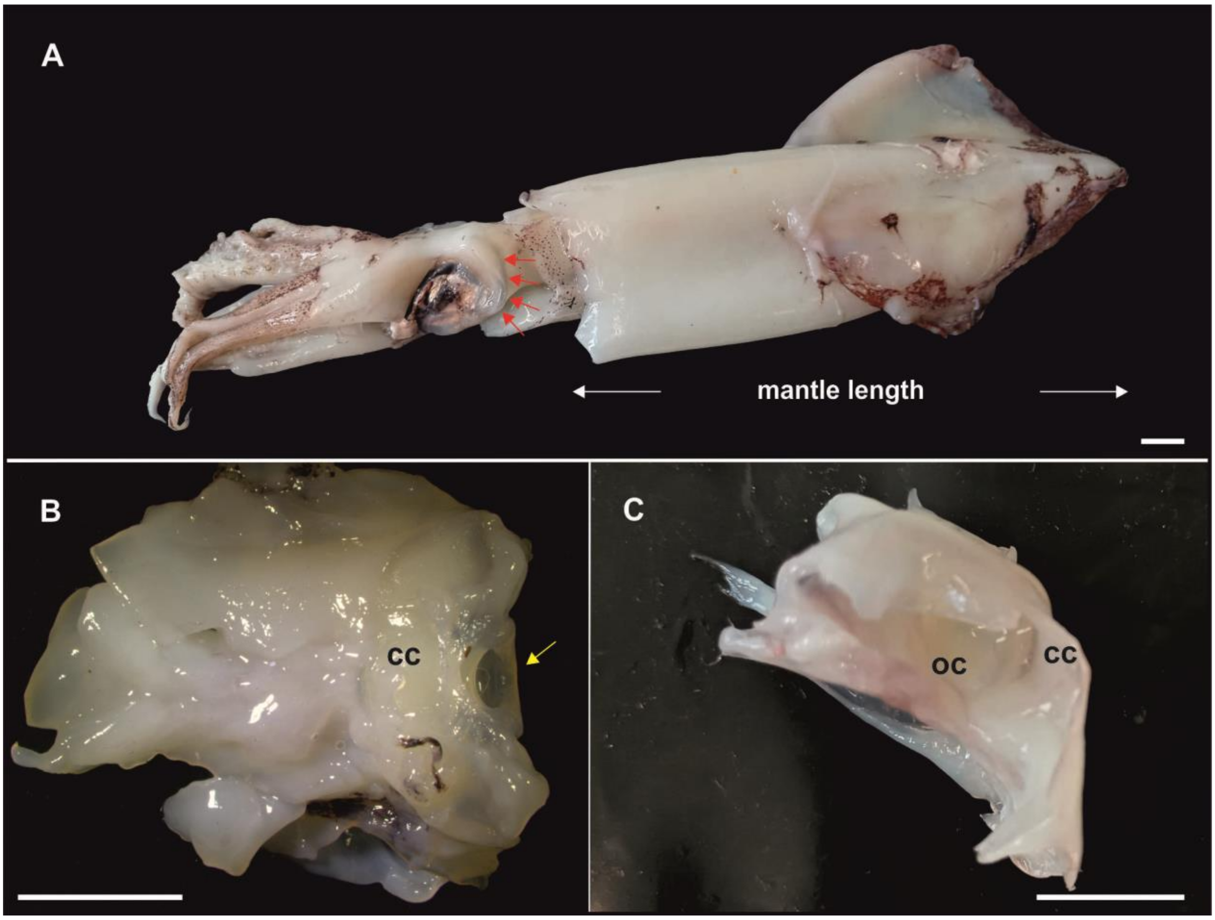

Supplement: S4 Fig — A. Specimen of Loligo vulgaris, red arrows marking position of cephalic cartilage. B. Well developed cephalic cartilage of Loligo vulgaris specimen from cranial view, yellow arrow pointing to opening for oesophagus. C Cephalic cartilage from lateral view, exhibiting ocular cartilage. cc cephalic cartilage; oc ocular cartilage. Scale bars 1 cm. (PDF) [file pone.0264595.s004.pdf]
