# Supplementary material for: Mineralized belemnoid cephalic cartilage from the late Triassic Polzberg Konservat-Lagerstätte (Austria)
Source: PLoS One. 2022 Apr 20;17(4):e0264595. doi: 10.1371/journal.pone.0264595 (PMC9020720; doi:10.1371/journal.pone.0264595)

Supporting Figure S9. Visualization of metrics, measured on specimens. h_c height of C structure; l_p length of processus; a_h height of arm cartilage. Scale bars 1 cm.

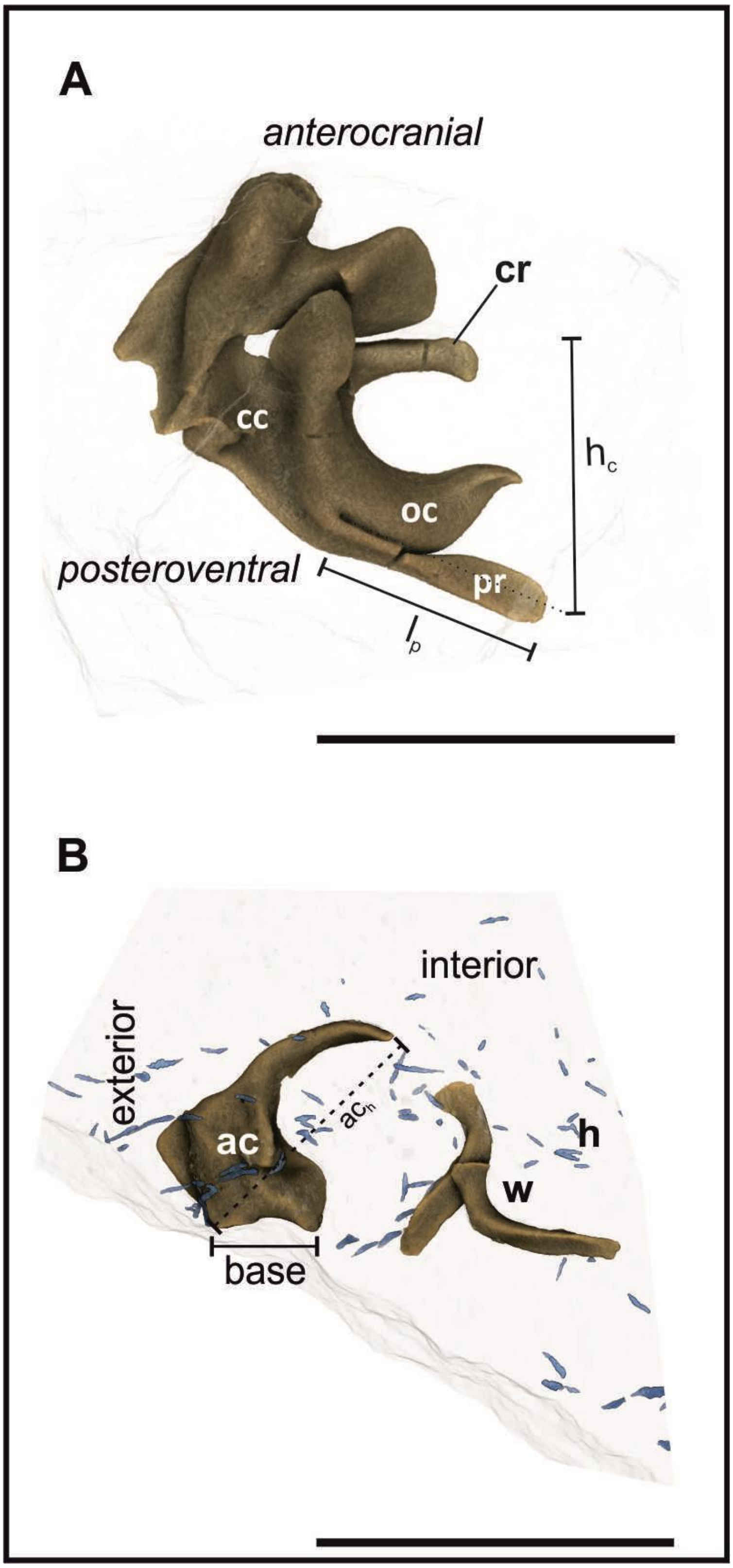

Supplement: S5 Fig — hc height of C structure; lp length of processus; mh height of megahook; mb base-length of megahook. Scale bars 1 cm. (PDF) [file pone.0264595.s005.pdf]
