# Supplementary material for: Mineralized belemnoid cephalic cartilage from the late Triassic Polzberg Konservat-Lagerstätte (Austria)
Source: PLoS One. 2022 Apr 20;17(4):e0264595. doi: 10.1371/journal.pone.0264595 (PMC9020720; doi:10.1371/journal.pone.0264595)
Supplement: S1 Table — The features of examined specimens and conducted methods, as well as associated belemnoid remains (phragmocone, proostracum, hooks) are given. The term indet. fossil in the table refers to here described black structures. Fifty-nine samples stem from Polzberg locality, seven from Rinngraben ravine near Cave del Predil (Julian Alps, Italy). Indicated are inventory numbers, locality, Type A or Type B fossil and/or wing, the applied methods (Micro-CT scanning resolution is given in brackets) and preserved belemnoid features. In recently collected specimens, the outcrop layer is given. NHMW corresponds to all inventory numbers except where GBA is given. Multiple elements on one slab are not separately mentioned;; PO Polzberg main section, ms measurements. (PDF) [file pone.0264595.s006.pdf]

Supporting Table S1. List of examined samples. The features of examined specimens and conducted methods, as well as associated belemnoid remains (phragmocone, proostracum, hooks) are given. The term indet. fossil in the table refers to here described black structures. Fifty-nine samples stem from the Polzberg locality, seven from Rinngraben ravine near Cave del Predil (Julian Alps, Italy). Indicated are inventory numbers, locality, Type A or Type B fossil and/or wing, the applied methods (Micro-CT scanning resolution is given in brackets) and preserved belemnoid features. In recently collected specimens, the outcrop layer is given. NHMW corresponds to all inventory numbers except where GBA is given. Multiple elements on one slab are not separately mentioned; PO Polzberg section, ms measurements.

	Inventory number	locality / layers	Type	applied method	features
1	GBA 2006/011/0003	Cave del Predil (Italy)	A	ms	phragmocone, proostracum, indet. fossil
2	GBA 2006/011/0012	Cave del Predil (Italy)	A	Micro-CT (27 µm)	phragmocone, proostracum, indet. fossil
3	GBA 2006/011/0020	Cave del Predil (Italy)	A	ms	hooks, phragmocone, proostracum, indet. fossil
4	GBA 2006/011/0028	Cave del Predil(Italy)	A	ms	phragmocone, proostracum, indet. fossil (negative)
5	GBA 2006/011/0041	Cave del Predil (Italy)	A	ms	indet. fossil
6	NHMW 2005z0005/0021	Cave del Predil (Italy)	A	ms	hooks, phragmocone, proostracum, indet. fossil
7	NHMW 2005z0005/0033	Cave del Predil (Italy)	A	ms	hooks, phragmocone, proostracum, indet. fossil
8	NHMW 2012/0117/0001	Polzberg (Lower Austria)	A	Micro-CT (27 µm), ms	indet. fossil
9	NHMW 2012/0117/0003	Polzberg (Lower Austria)	A	ms	indet. fossil
10	NHMW 2012/0117/0006	Polzberg (Lower Austria)	A	Micro-CT (22 µm), ms	indet. fossil
11	NHMW 2012/0117/0007	Polzberg (Lower Austria)	B	ms	indet. fossil
12	NHMW 2012/0117/0009	Polzberg (Lower Austria)	A	Micro-CT (16.5 µm), ms	indet. fossil
13	NHMW 2012/0117/0010	Polzberg (Lower Austria)	A	ms	indet. fossil

14	NHMW 2012/0117/0011	Polzberg (Lower Austria)	A, w	Micro-CT (15.5 µm), ms	hooks, indet. fossil
15	NHMW 2012/0117/0012	Polzberg (Lower Austria)	B	Micro-CT (23 µm), ms	indet. fossil
16	NHMW 2012/0117/0013	Polzberg (Lower Austria)	A, w	ms	indet. fossil
17	NHMW 2012/0117/0014	Polzberg (Lower Austria)	A	Micro-CT (16.5 µm), ms	indet. fossil
18	NHMW 2012/0117/0015	Polzberg (Lower Austria)	A	REM, ms	hooks, indet. fossil
19	NHMW 2012/0117/0016	Polzberg (Lower Austria)	A	ms	indet. fossil
20	NHMW 2012/0117/0018	Polzberg (Lower Austria)	A, w	ms	hooks, indet. fossil
21	NHMW 2012/0117/0019	Polzberg (Lower Austria)	A	ms	indet. fossil
22	NHMW 2012/0117/0021	Polzberg (Lower Austria)	A	ms	indet. fossil
23	NHMW 2012/0117/0024	Polzberg (Lower Austria)	A	Microprobe, REM, Thin-section, ms	indet. fossil
24	NHMW 2012/0117/0025	Polzberg (Lower Austria)	A, w	Micro-CT (33.5µm), ms	hooks, proostracum, indet. fossil
25	NHMW 2012/0117/0026	Polzberg (Lower Austria)	B	ms	indet. fossil
26	NHMW 2012/0117/0028	Polzberg (Lower Austria)	A	Micro-CT (15 µm), ms	indet. fossil
27	NHMW 2012/0117/0031	Polzberg (Lower Austria)	A	ms	indet. fossil
28	NHMW 2012/0117/0032	Polzberg (Lower Austria)	w	ms	hooks, indet. fossil
29	NHMW 2012/0228/1718	Polzberg (Lower Austria)	B	ms	indet. fossil
30	NHMW 2012/0228/1719	Polzberg (Lower Austria)	B	ms	indet. fossil
31	NHMW 2021/0001/0002	Polzberg (Lower Austria)	A, w	Micro-CT (20µm), ms	hooks, indet. fossil
32	NHMW 2021/0016/0273	Polzberg (Lower Austria)	A	ms	indet. fossil
33	NHMW 2021/0016/0275	Polzberg (Lower Austria)	w	ms	indet. fossil
34	NHMW 2021/0016/0396	Polzberg (Lower Austria)	B	ms	indet. fossil
35	NHMW 2021/0016/0397	Polzberg (Lower Austria)		ms	indet. fossil
36	NHMW 2021/0016/0398	Polzberg (Lower Austria)		ms	indet. fossil
37	NHMW 2021/0016/0399	Polzberg (Lower Austria)		ms	indet. fossil
38	NHMW 2021/0016/0400	Polzberg (Lower Austria) / PO -50–0 cm	A	ms	hooks, proostracum, indet. fossil
39	NHMW 2021/0123/0003	Polzberg (Lower Austria)	A	ms	indet. fossil
40	NHMW 2021/0123/0010	Polzberg (Lower Austria)	B	ms	indet. fossil
41	NHMW 2021/0123/0011	Polzberg (Lower Austria) / PO -50–0 cm	A	Raman, ms	indet. fossil
42	NHMW 2021/0123/0013	Polzberg (Lower Austria) / PO -50–0 cm	A, w	Micro-CT (25.5 µm), ms	hooks, indet. fossil

43	NHMW 2021/0123/0044	Polzberg (Lower Austria) / PO 60–80 cm	B	ms	indet. fossil
44	NHMW 2021/0123/0047	Polzberg (Lower Austria) / PO 80–100 cm	A	ms	indet. fossil
45	NHMW 2021/0123/0048	Polzberg (Lower Austria) / PO 100–120 cm	A	ms	indet. fossil
46	NHMW 2021/0123/0049	Polzberg (Lower Austria) / PO 140-160 cm	A	ms	indet. fossil
47	NHMW 2021/0123/0050	Polzberg (Lower Austria) / PO -50–0 cm	A	ms	indet. fossil
48	NHMW 2021/0123/0053	Polzberg (Lower Austria) / PO -50–0 cm	A	ms	indet. fossil
49	NHMW 2021/0123/0054	Polzberg (Lower Austria) / PO -50–0 cm	A	ms	indet. fossil
50	NHMW 2021/0123/0055	Polzberg (Lower Austria) / PO -50–0 cm	B	ms	indet. fossil
51	NHMW 2021/0123/0057	Polzberg (Lower Austria) / PO 60–80 cm	A, w	ms	hooks, indet. fossil
52	NHMW 2021/0123/0070	Polzberg (Lower Austria) / PO -50–0 cm	A	ms	indet. fossil
53	NHMW 2021/0123/0071	Polzberg (Lower Austria) / PO -50–0 cm	w	ms	indet. fossil
54	NHMW 2021/0123/0072	Polzberg (Lower Austria) / PO -50–0 cm	A	ms	indet. fossil
55	NHMW 2021/0123/0074	Polzberg (Lower Austria) / PO 60–80 cm	A, w	ms	indet. fossil
56	NHMW 2021/0123/0129	Polzberg (Lower Austria) / PO -50–0 cm	A, w	ms	hooks, indet. fossil
57	NHMW 2021/0123/0130	Polzberg (Lower Austria)	A	ms	indet. fossil
58	NHMW 2021/0123/0165	Polzberg (Lower Austria	A	ms	Indet fossil
59	NHMW 2021/0123/0166	Polzberg (Lower Austria	A	ms	Indet fossil
60	NHMW 2021/0124/0001	Polzberg (Lower Austria)	A	ms	indet. fossil
61	NHMW 2021/0124/0002	Polzberg (Lower Austria)	B	ms	indet. fossil
62	NHMW 2021/0124/0003	Polzberg (Lower Austria)	B,w	Micro-CT (17.5 µm), ms	hooks, indet. fossil
63	NHMW 2021/0124/0004	Polzberg (Lower Austria)	B	Micro-CT (15.5 µm), ms	indet. fossil
64	NHMW 2021/0124/0006	Polzberg (Lower Austria)	A	ms	hooks, indet. fossil

65	NHMW 2021/0124/0010	Polzberg (Lower Austria)	B	ms	indet. fossil
66	NHMW 2021/0124/0077	Polzberg (Lower Austria)	A, w	ms	indet. fossil
