# Supplementary material for: Mineralized belemnoid cephalic cartilage from the late Triassic Polzberg Konservat-Lagerstätte (Austria)
Source: PLoS One. 2022 Apr 20;17(4):e0264595. doi: 10.1371/journal.pone.0264595 (PMC9020720; doi:10.1371/journal.pone.0264595)
Supplement: S2 Table — fossl full length of visible phragmocone-proostracum; pw width of phragmocone; prol length proostracum; d distance last field of proostracum–fossil structure remarks. All measurements in mm. Only specimens listed, where measurements were possible. (PDF) [file pone.0264595.s007.pdf]

Supporting Table S2. Metrics of black fossil structures in association with coleoid remains. $foss_l$ full length of visible phragmocone-proostracum; p_w width of phragmocone; pro_l length proostracum; d distance last field of proostracum – fossil structure remarks. All measurements in mm. Only specimens listed, where measurements were possible.

Inventory number	Type	$foss_l$	p_w	pro_l	d	remarks	locality
GBA 2006/011/0003	A	58.03	23.23	40.74	26.48		Cave del Predil (Italy)
GBA 2006/011/0028	A	67.26	25.62	40.08	29.91	only negative of black fossil	Cave del Predil (Italy)
GBA 2006/011/0012	A	39.59	13.55	31.43	26.59	two black knobs between proostracum and enigmatic fossil	Cave del Predil (Italy)
GBA 2006/011/0020	A	70.34	28.52	39.89	31.80		Cave del Predil (Italy)
NHMW 2012/0117/0025	A	-	-	-	20.03	only part of last proostracum field preserved; microhooks present	Polzberg (Lower Austria)
NHMW 2005z0005/0033	A	58.59	25.85	35.38	28.25	microhooks present	Cave del Predil (Italy)
NHMW 2005z0005/0021	A	47.30	14.92	28.59	19.13	microhooks present	Polzberg (Lower Austria)
NHMW 2021/0016/0400	A	63.05	40.11	63.05	23.95	only part of proostracum, microhooks present	Polzberg (Lower Austria)
