# Supplementary material for: Mineralized belemnoid cephalic cartilage from the late Triassic Polzberg Konservat-Lagerstätte (Austria)
Source: PLoS One. 2022 Apr 20;17(4):e0264595. doi: 10.1371/journal.pone.0264595 (PMC9020720; doi:10.1371/journal.pone.0264595)
Supplement: S3 Table — (PDF) [file pone.0264595.s008.pdf]

Supporting Table S5. Micro-CT Scan parameters for fossil specimens.

Specimen	Resolution (µm)	kV	Type	Exposure time (ms)	Pre-filter
GBA 2006/011/0012	27.0	150	270	1400	0.75 mm copper
NHMW 2012/0117/0025	33.5	150	270	1400	0.75 mm copper
NHMW 2021/0124/0003	17.5	150	180	2000	0.75 mm copper
NHMW 2021/0124/0004	15.5	150	180	2000	0.75 mm copper
NHMW 2021/0016/0399	25.5	150	280	1700	1.00 mm copper
NHMW 2012/0117/0001	27.0	150	280	1700	1.00 mm copper
NHMW 2012/0117/0006	22.0	150	240	1400	0.75 mm copper
NHMW 2012/0117/0009	16.5	150	180	2000	0.75 mm copper
NHMW 2012/0117/0011	15.5	150	180	2000	0.75 mm copper
NHMW 2012/0117/0012	23.0	150	270	1400	0.75 mm copper
NHMW 2012/0117/0014	16.5	150	180	2000	0.75 mm copper
NHMW 2012/0117/0028	15.0	150	180	2000	0.75 mm copper
NHMW 2021/0001/0002	20.0	150	210	1700	0.75 mm copper
