# Supplementary material for: Mineralized belemnoid cephalic cartilage from the late Triassic Polzberg Konservat-Lagerstätte (Austria)
Source: PLoS One. 2022 Apr 20;17(4):e0264595. doi: 10.1371/journal.pone.0264595 (PMC9020720; doi:10.1371/journal.pone.0264595)
Supplement: S4 Table — Each specimen of each sample is listed here measured separately; hc height of C structure; lp length of processus; lw length of wing; wb base-length of wing element; ach height of arm cartilage; acb base-length of arm cartilage; all measurements in mm. Elements could not be measured in GBA specimens and are therefore not listed. Only specimens listed, where measurements were possible. (PDF) [file pone.0264595.s009.pdf]

Supporting Table S10. Measurements conducted on fossil specimens. Each specimen of each sample is listed here measured separately; h_c height of C structure; l_p length of processus; l_w length of wing; w_b base-length of wing element; ac_h height of arm cartilage; ac_b base-length of arm cartilage; all measurements in mm. Elements could not be measured in GBA specimens and are therefore not listed. Only specimens listed, where measurements were possible.

	Inv.- number	Type	h_c	l_p	l_w	w_b	ac_h	ac_b
1	NHMW 2005z0005/0021	A	3.52	3.76				
2	NHMW 2005z0005/0033	A						
3	NHMW 2012/0117/0001	A	7.62	6.37				
4	NHMW 2012/0117/0003	A	6.59					
5	NHMW 2012/0117/0006	A	4.92	6.75				
6	NHMW 2012/0117/0006	A	5.19					
7	NHMW 2012/0117/0007	B					8.67	5.38
8	NHMW 2012/0117/0009	A	5.78	6.28				
9	NHMW 2012/0117/0010	A						
10	NHMW 2012/0117/0011	A, w	6.51					
11	NHMW 2012/0117/0012	B					6.24	5.56
12	NHMW 2012/0117/0012	B					6.58	5.50
13	NHMW 2012/0117/0012	B					6.68	5.54
14	NHMW 2012/0117/0013	A	7.37					
15	NHMW 2012/0117/0013	w			7.18	4.67		
16	NHMW 2012/0117/0014	A	6.60	7.14				
17	NHMW 2012/0117/0015	A	6.66	4.74				
18	NHMW 2012/0117/0015	w			7.62			
19	NHMW 2012/0117/0016	A		8.87				
20	NHMW 2012/0117/0018	A	4.68					
21	NHMW 2012/0117/0018	w			7.09	3.94		
22	NHMW 2012/0117/0019	A	5.71					
23	NHMW 2012/0117/0021	A						
24	NHMW 2012/0117/0024	A						

25	NHMW 2012/0117/0025	A	6.47					
26	NHMW 2012/0117/0025	w			8.90	4.41		
27	NHMW 2012/0117/0026	B					7.68	5.20
28	NHMW 2012/0117/0028	A	7.47					
29	NHMW 2012/0117/0028	A		7.03				
30	NHMW 2012/0117/0031	A						
31	NHMW 2012/0117/0032	w			8.14	4.60		
32	NHMW 2012/0228/1718	B					6.75	5.17
33	NHMW 2012/0228/1719	B			7.47	4.37		
34	NHMW 2021/0001/0002	A	8.08	8.10				
35	NHMW 2021/0001/0002	A, w	6,98		8.81	5.48		
36	NHMW 2021/0016/0273	A	5.15	6.35				
37	NHMW 2021/0016/0275	w			7.69	4.49		
38	NHMW 2021/0016/0396	B					6.62	3.32
39	NHMW 2021/0016/0397	A	7.97					
40	NHMW 2021/0016/0398	A	6.70					
41	NHMW 2021/0016/0399	A, w	6.89		8.00	3.64		
42	NHMW 2021/0016/0400	A	6.30					
43	NHMW 2021/0123/0003	A						
44	NHMW 2021/0123/0010	B					10.58	5,64
45	NHMW 2021/0123/0011	A	9.99					
46	NHMW 2021/0123/0013	A, w						
47	NHMW 2021/0123/0044	B						
48	NHMW 2021/0123/0047	A	6.8	7.65				
49	NHMW 2021/0123/0048	A	7.97	5.86				
50	NHMW 2021/0123/0049	A	8.49					
51	NHMW 2021/0123/0050	A	6.92	7.94				
52	NHMW 2021/0123/0050	A	7.27	6.36				
53	NHMW 2021/0123/0053	A						
54	NHMW 2021/0123/0054	A						
55	NHMW 2021/0123/0055	B					9.04	6.60
56	NHMW 2021/0123/0057	A, w	8.02	9.42				
57	NHMW 2021/0123/0070	A	5.56	5.55				
58	NHMW 2021/0123/0070	A	6.15					

59	NHMW 2021/0123/0071	w			6.35	3.63		
60	NHMW 2021/0123/0072	A		6.06				
61	NHMW 2021/0123/0074	A, w	7.12	5.89				
62	NHMW 2021/0123/0074	A						
63	NHMW 2021/0123/0129	A						
64	NHMW 2021/0123/0129	w			6.46	3.50		
65	NHMW 2021/0123/0130	A						
66	NHMW 2021/0123/0165	A	7.78					
67	NHMW 2021/0123/0166	A						
68	NHMW 2021/0124/0001	A	8.39	8.17				
69	NHMW 2021/0124/0002	B					7.86	4.24
70	NHMW 2021/0124/0002	w			6.35			
71	NHMW 2021/0124/0003	B					8.06	6.14
72	NHMW 2021/0124/0003	B					7.44	6.12
73	NHMW 2021/0124/0003	w			6.13			
74	NHMW 2021/0124/0003	w			5.01			
75	NHMW 2021/0124/0003	B					8.51	5.97
76	NHMW 2021/0124/0004	B					9.38	6.04
77	NHMW 2021/0124/0004	B					8.16	4.98
78	NHMW 2021/0124/0006	A	6.26	6.01				
79	NHMW 2021/0124/0010	B					10.50	7.00
80	NHMW 2021/0124/0077	A, w	6.51		8.45	4.90		
81	NHMW 2021/0124/0077	A	6.50					
