# Supplementary material for: Mineralized belemnoid cephalic cartilage from the late Triassic Polzberg Konservat-Lagerstätte (Austria)
Source: PLoS One. 2022 Apr 20;17(4):e0264595. doi: 10.1371/journal.pone.0264595 (PMC9020720; doi:10.1371/journal.pone.0264595)
Supplement: S5 Table — (PDF) [file pone.0264595.s010.pdf]

Supporting Table S11. Volumes of fossil specimens, obtained from Micro-CT data.

Inv.- number	Voxelsize (µm)	Volume (mm³)
GBA 2006/011/0012	44.00	234.26
NHMW 2012/0117/0025	33.50	31.82
NHMW 2021/0001/0002	20.00	253.95
NHMW 2021/0124/0003	17.50	32.76
NHMW 2021/0124/0004	15.50	26.13
NHMW 2021/0016/0399	25.50	120.49
NHMW 2012/0117/0001	27.00	91.12
NHMW 2012/0117/0006	22.00	68.86
NHMW 2012/0117/0009	16.50	53.40
NHMW 2012/0117/0011	15.50	49.58
NHMW 2012/0117/0012	23.00	49.00
NHMW 2012/0117/0014	16.50	60.09
NHMW 2012/0117/0028	15.00	73.33
