# Supplementary material for: Mineralized belemnoid cephalic cartilage from the late Triassic Polzberg Konservat-Lagerstätte (Austria)
Source: PLoS One. 2022 Apr 20;17(4):e0264595. doi: 10.1371/journal.pone.0264595 (PMC9020720; doi:10.1371/journal.pone.0264595)
Supplement: S1 Data — (PDF) [file pone.0264595.s011.pdf]

Supporting Data S6. Microprobe report for sample NHMW 2012/0117/0024.

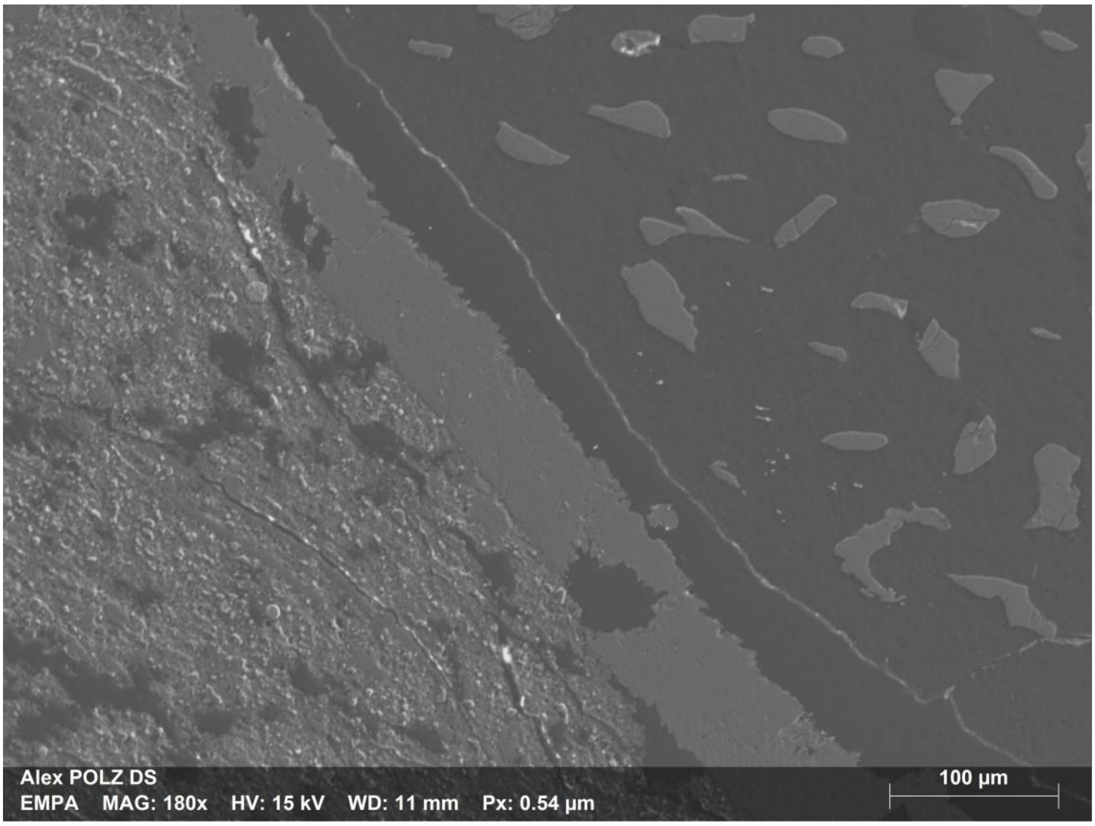

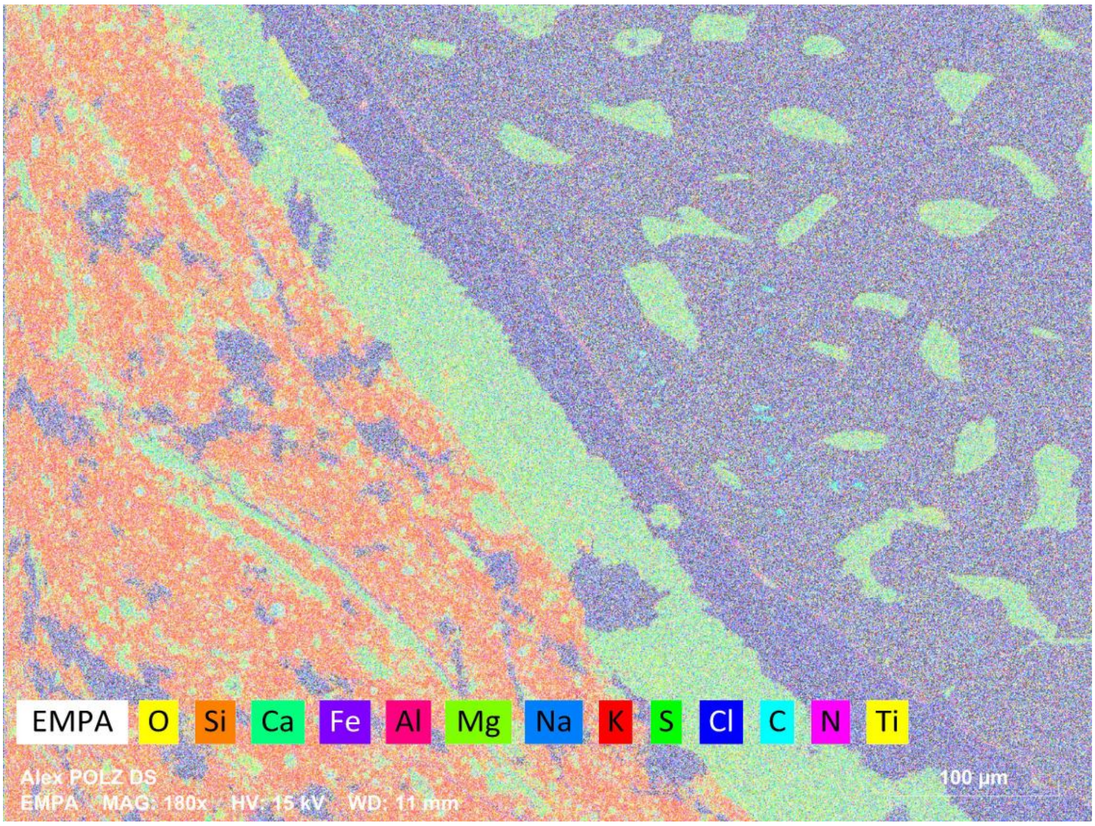

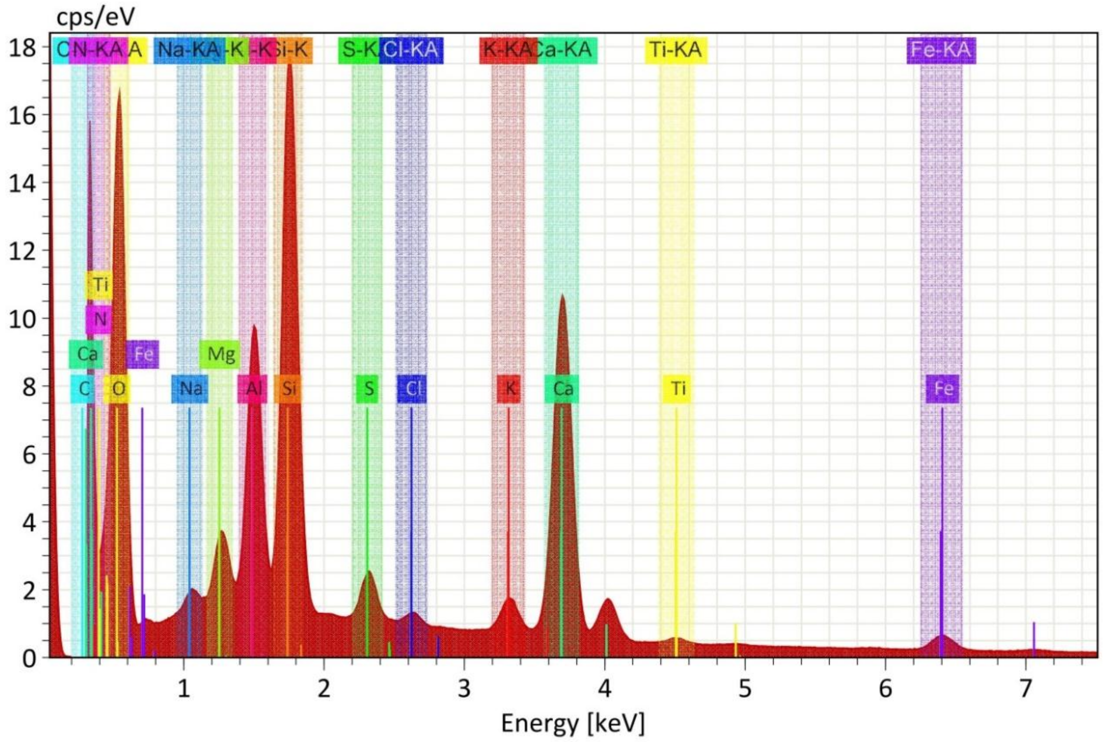
cps/eV
Energy [keV]
N-KA
Na-KA
Si-K
S-K
Cl-KA
K-KA
Ca-KA
Ti-KA
Fe-KA
Ti
N
Ca
Fe
Mg
C
O
Na
Al
Si
S
Cl
K
Ca
Ti
Fe

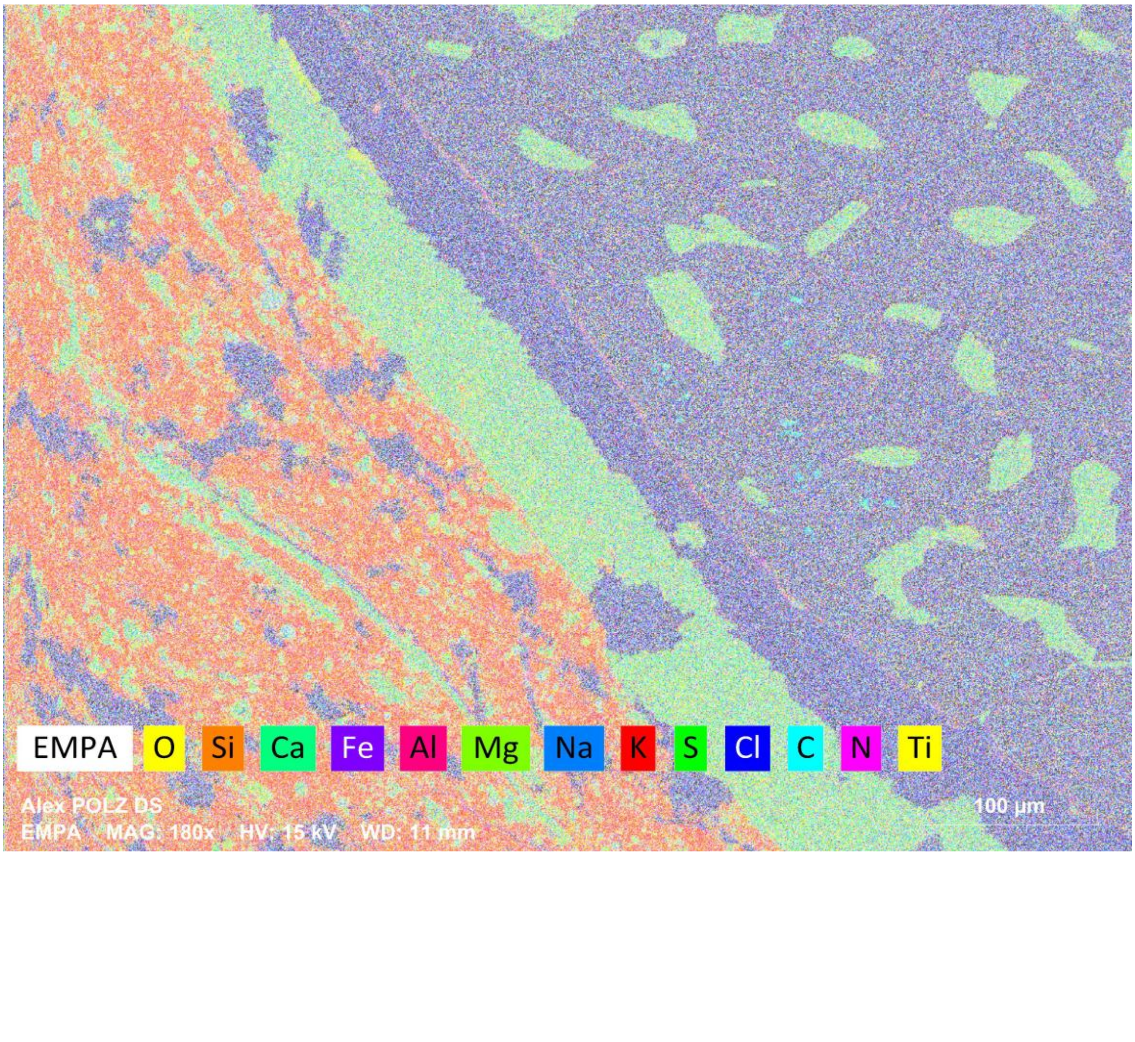
EMPA
O
Si
Ca
Fe
Al
Mg
Na
K
S
Cl
C
N
Ti
Alex POLZ DS
EMPA MAG: 180x HV: 15 kV WD: 11 mm
100 µm

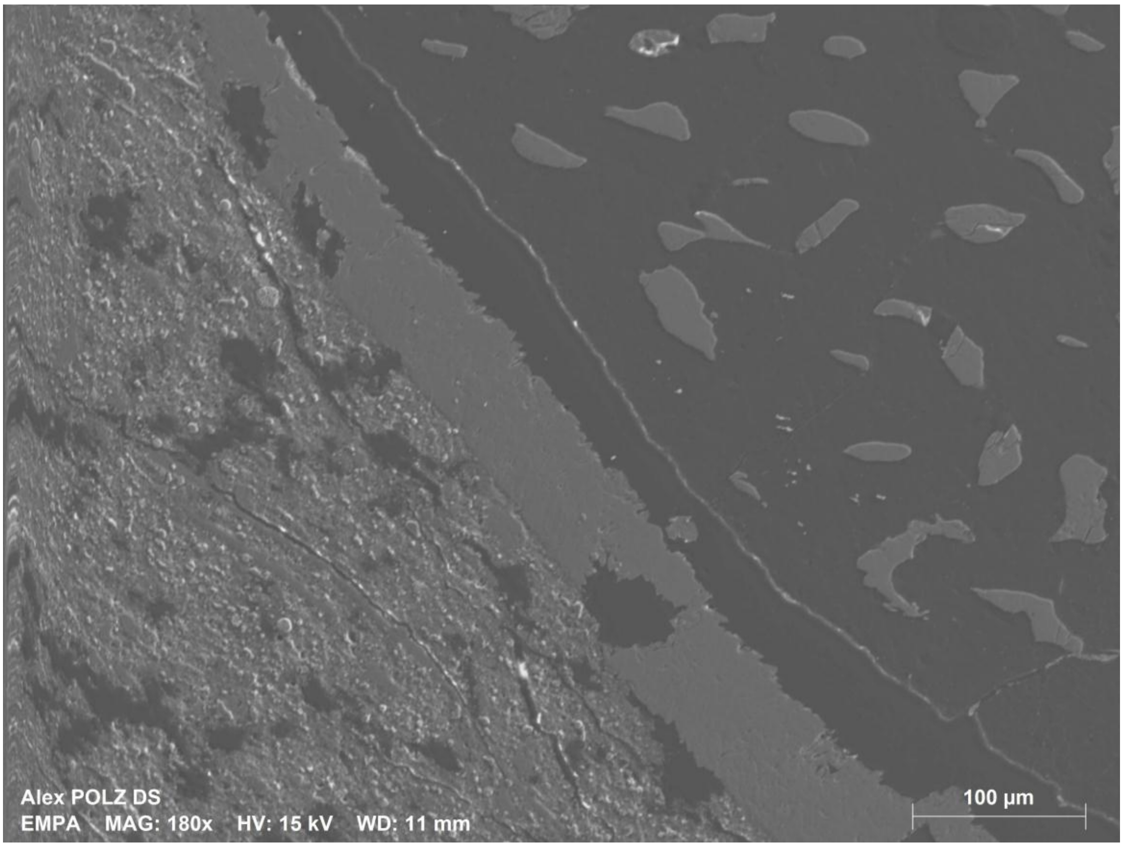
Alex POLZ DS
EMPA MAG: 180x HV: 15 kV WD: 11 mm
100 µm

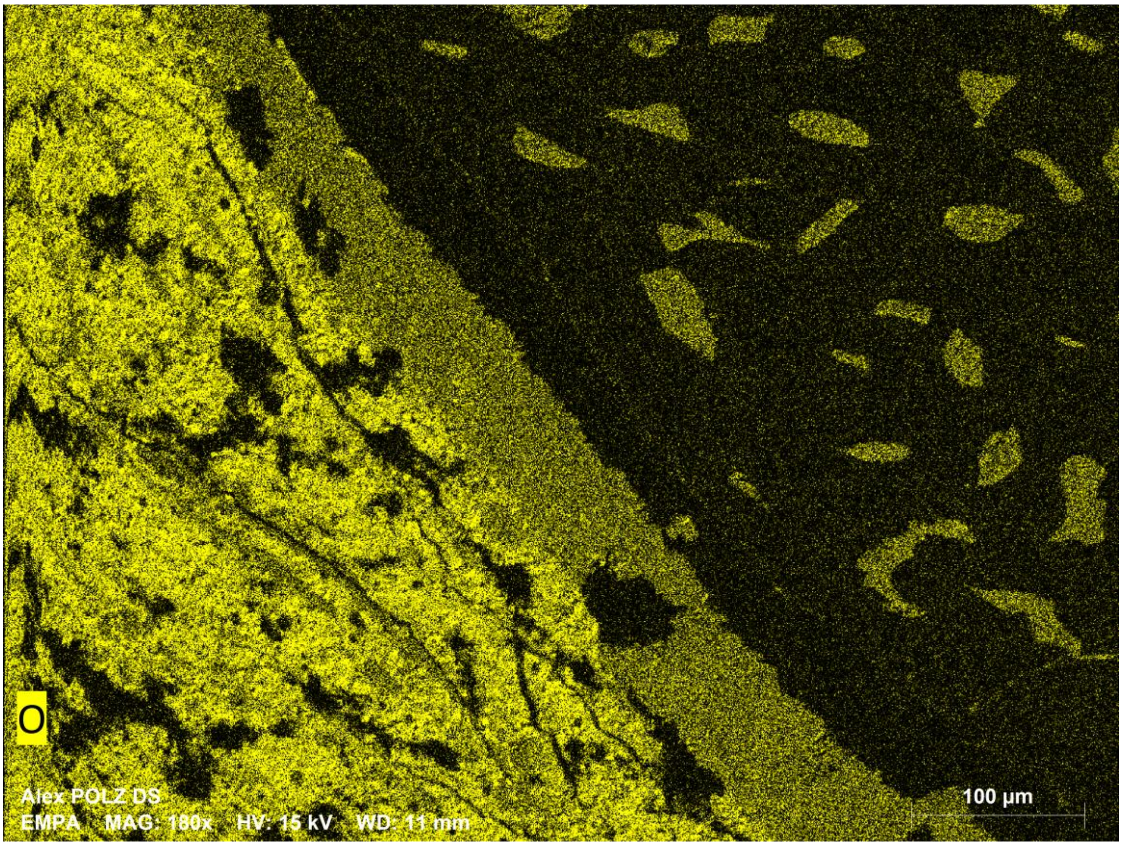
O
Alex POLZ DS
EMPA MAG: 180x HV: 15 kV WD: 11 mm
100 µm

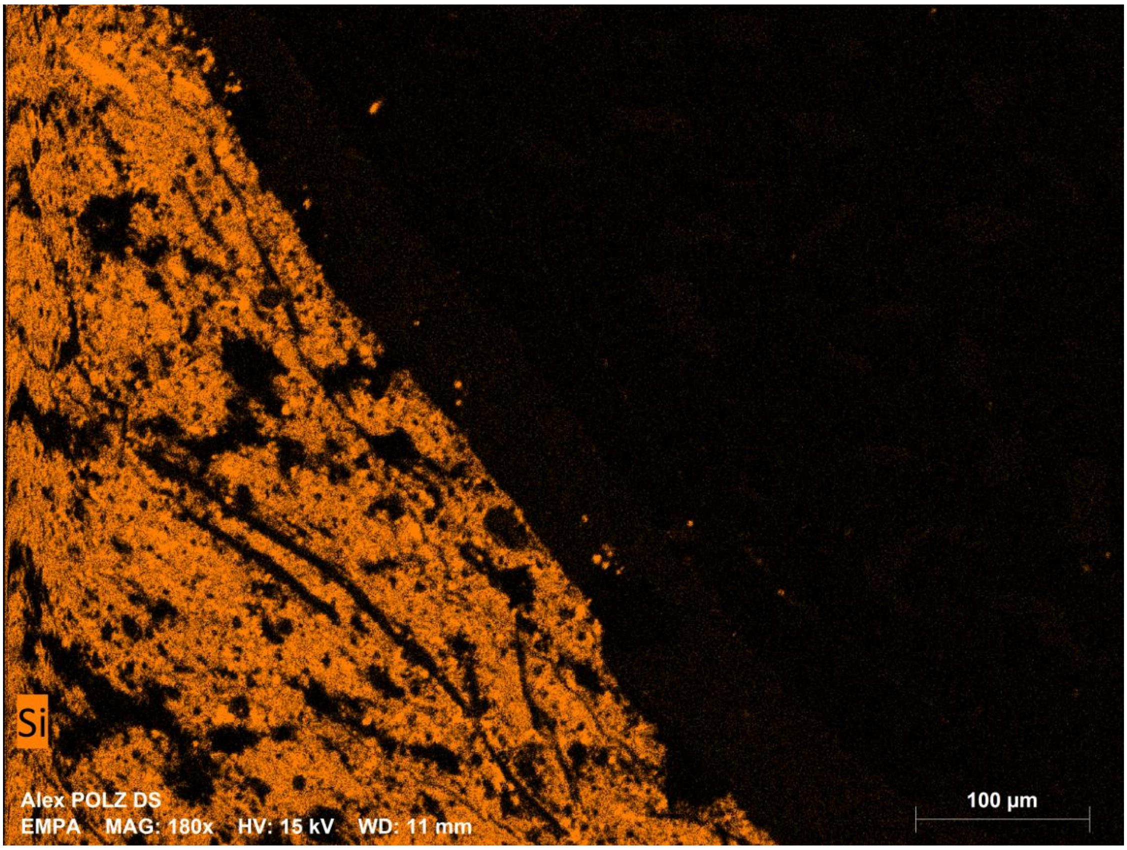
Si
Alex POLZ DS
EMPA MAG: 180x HV: 15 kV WD: 11 mm
100 µm

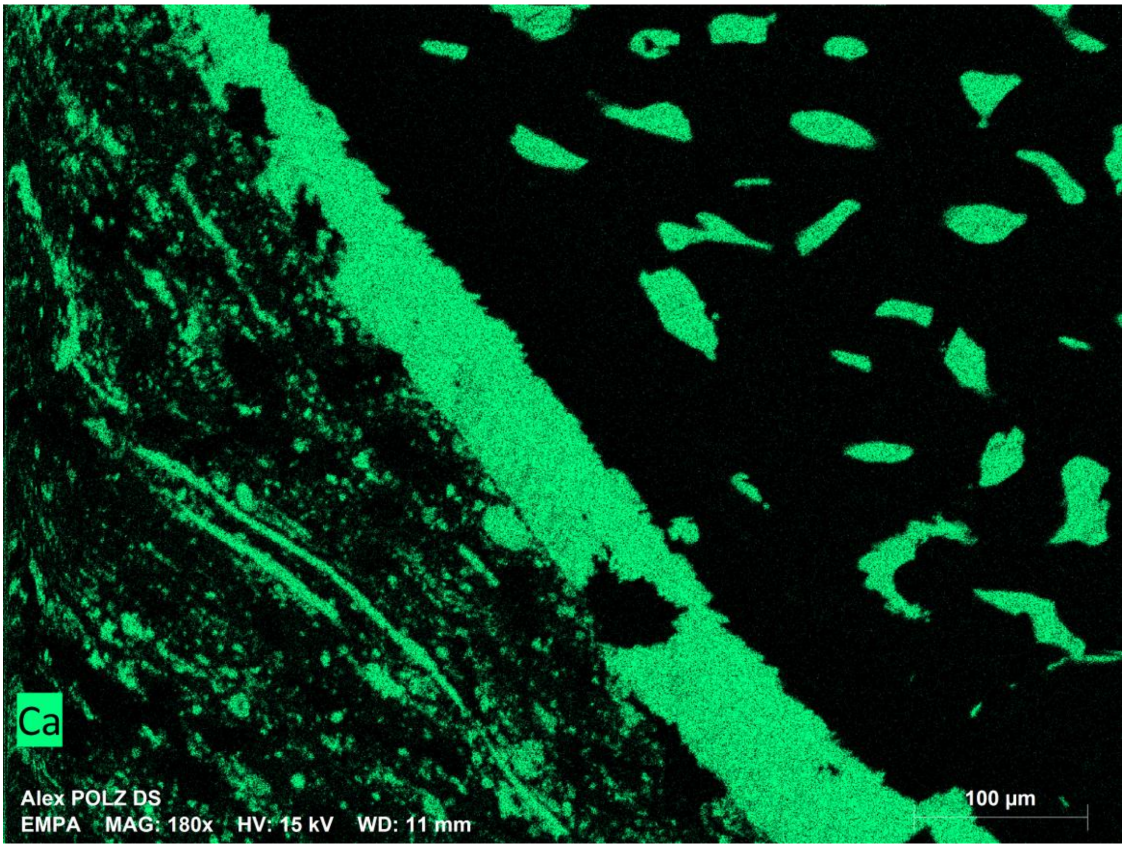
Ca
Alex POLZ DS
EMPA MAG: 180x HV: 15 kV WD: 11 mm
100 µm

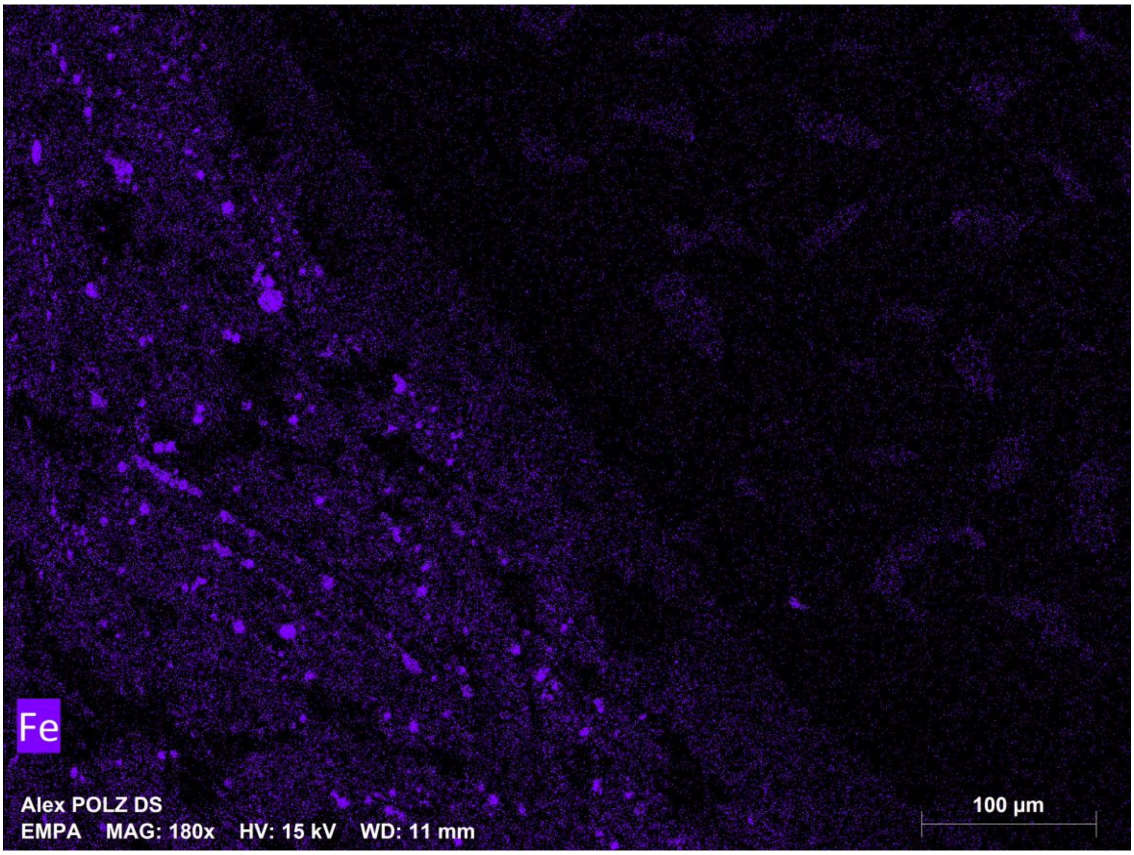
Fe
Alex POLZ DS
EMPA MAG: 180x HV: 15 kV WD: 11 mm
100 µm

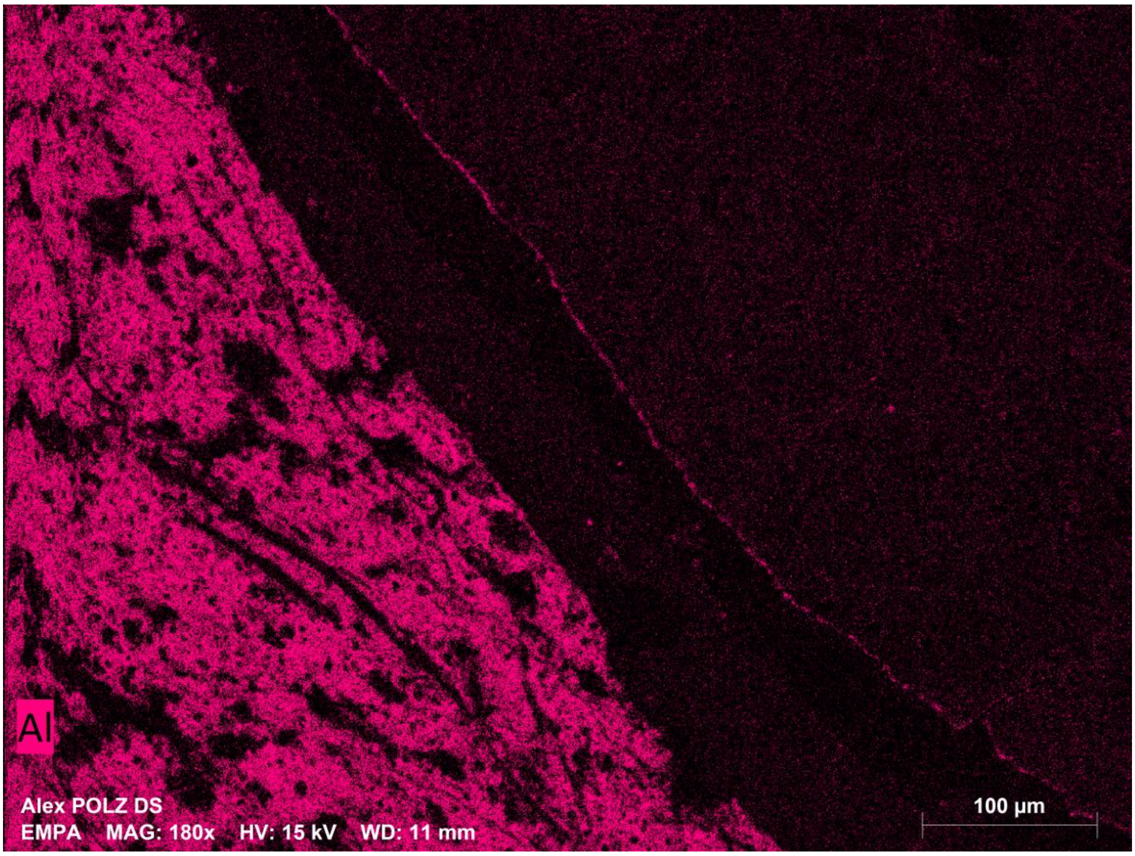
Al
Alex POLZ DS
EMPA MAG: 180x HV: 15 kV WD: 11 mm
100 µm

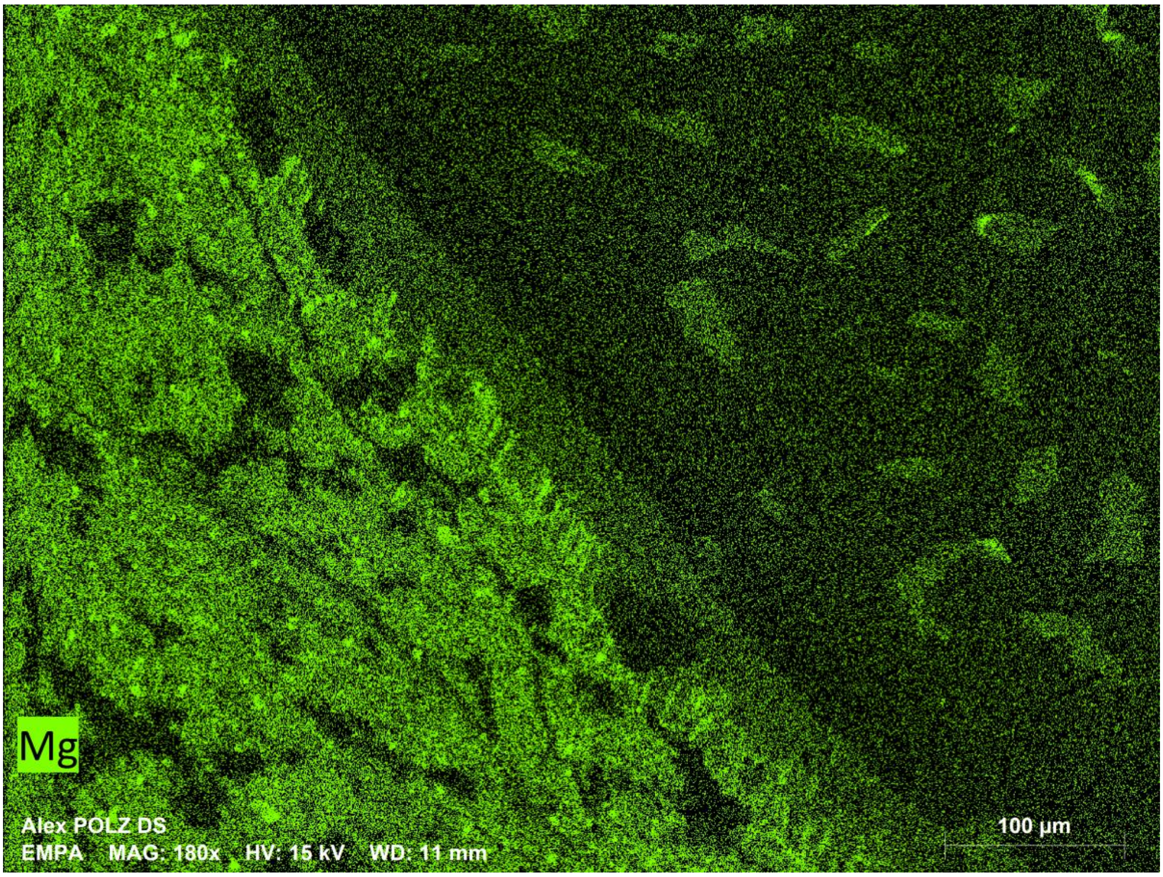
Mg
Alex POLZ DS
EMPA MAG: 180x HV: 15 kV WD: 11 mm
100 µm

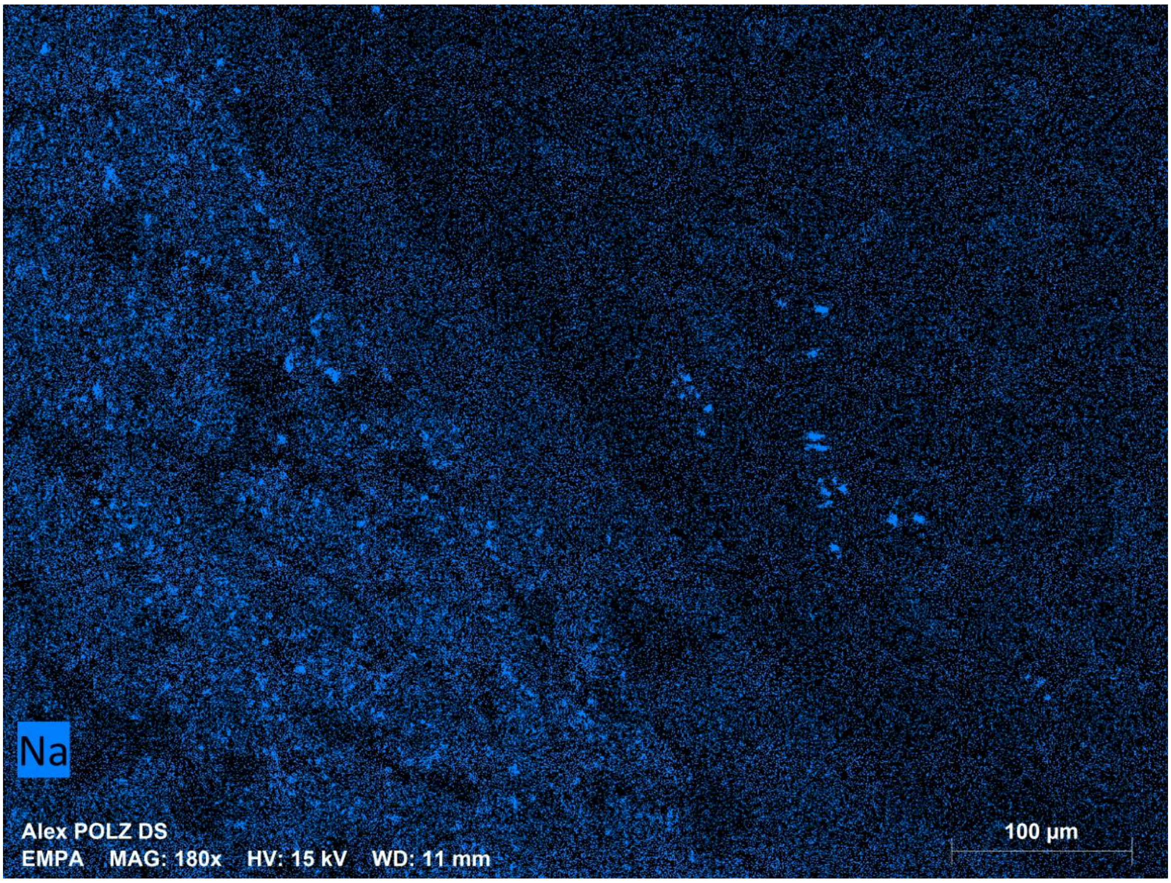
Na
Alex POLZ DS
EMPA MAG: 180x HV: 15 kV WD: 11 mm
100 µm

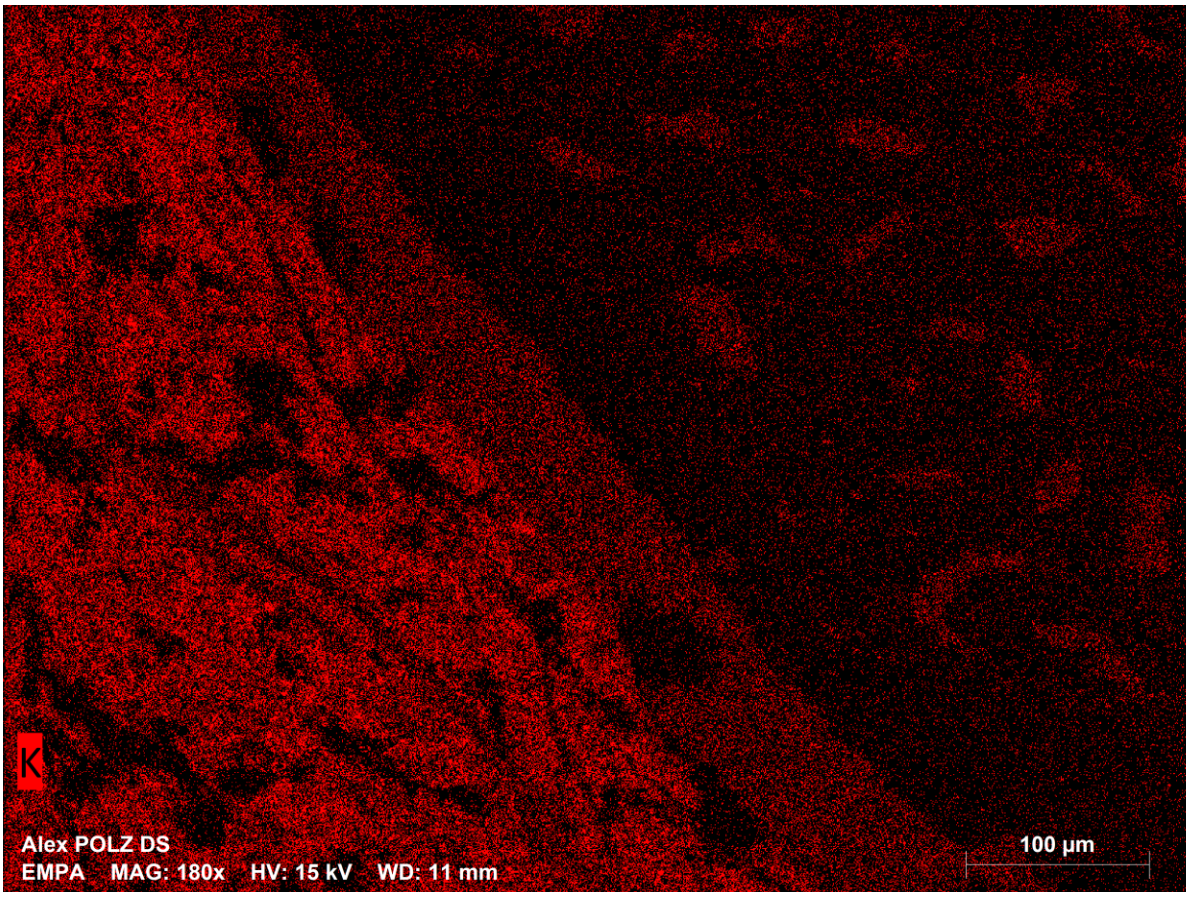
K
Alex POLZ DS
EMPA MAG: 180x HV: 15 kV WD: 11 mm
100 µm

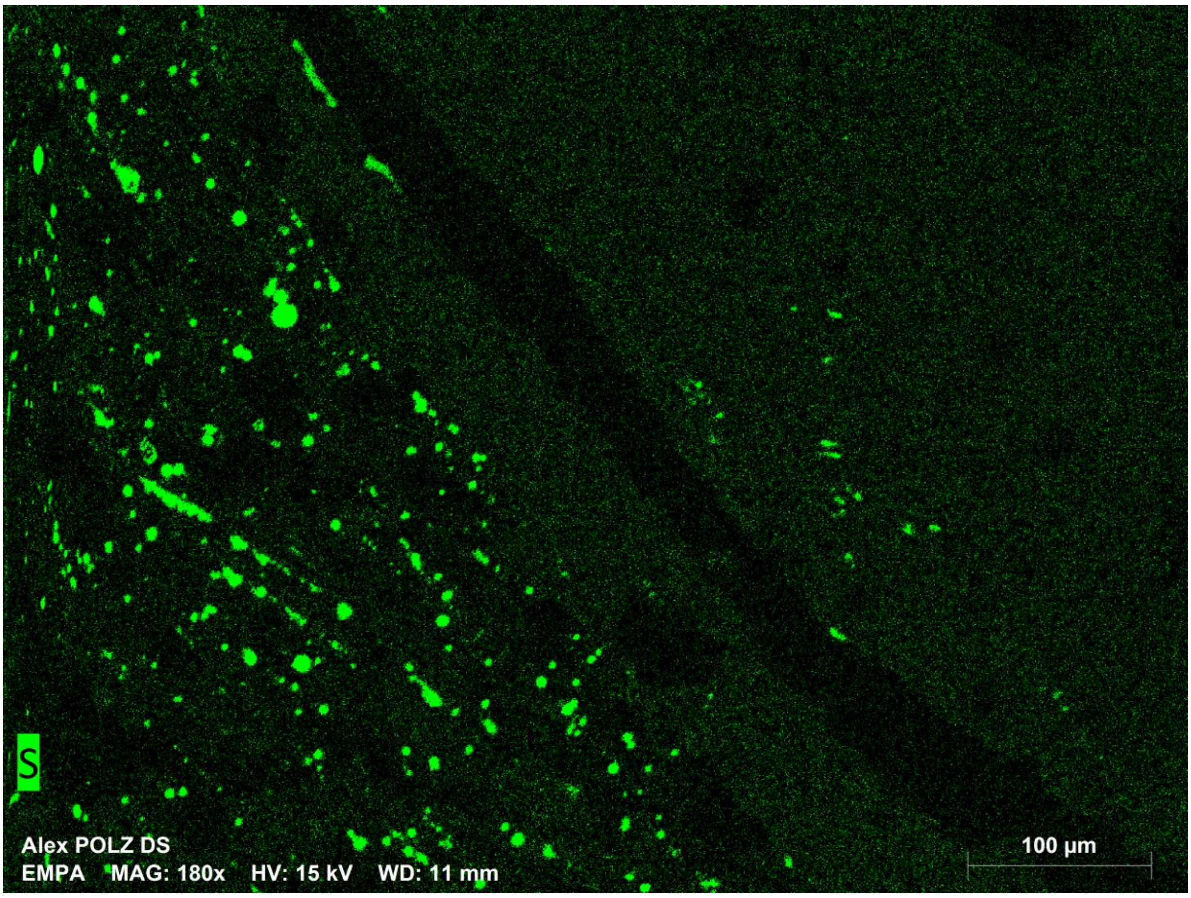
S
Alex POLZ DS
EMPA MAG: 180x HV: 15 kV WD: 11 mm
100 µm

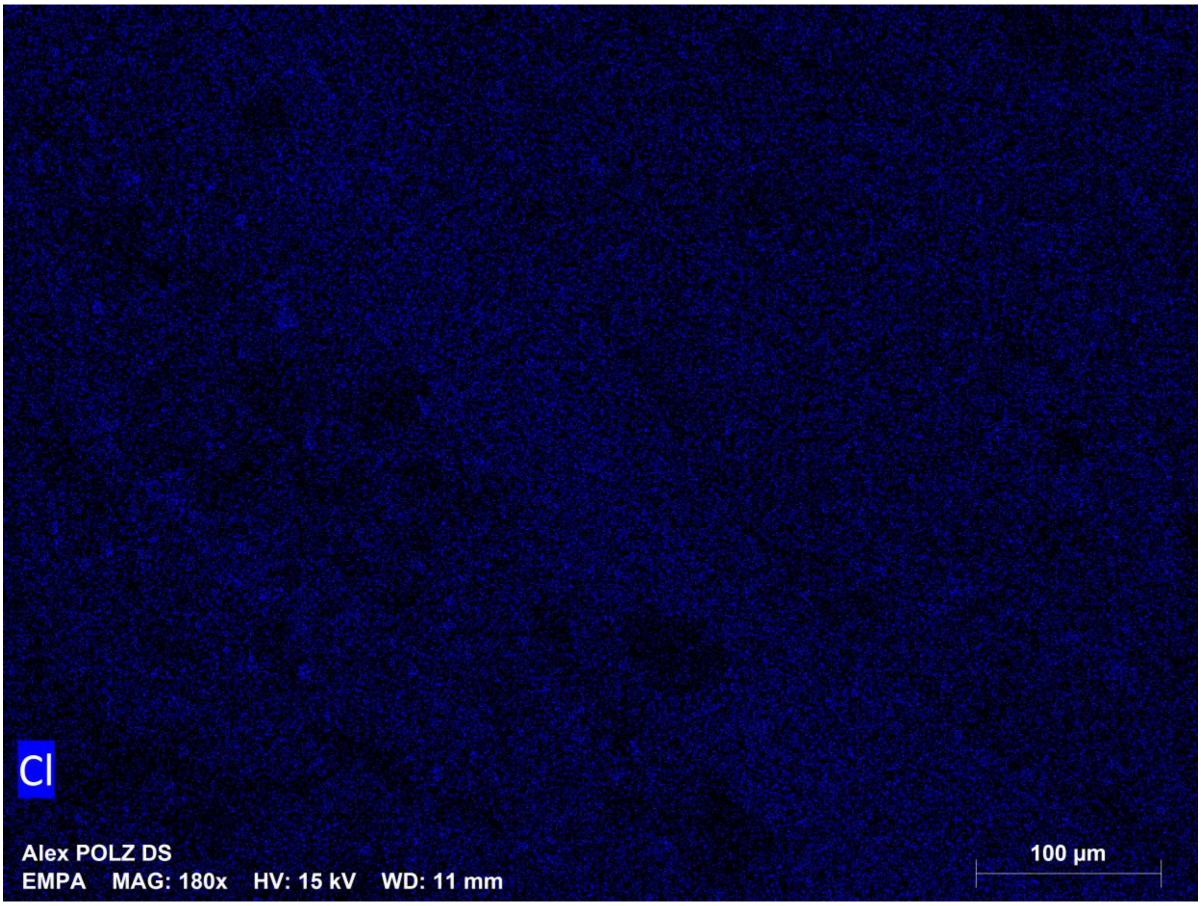

Cl
Alex POLZ DS
EMPA MAG: 180x HV: 15 kV WD: 11 mm
100 µm

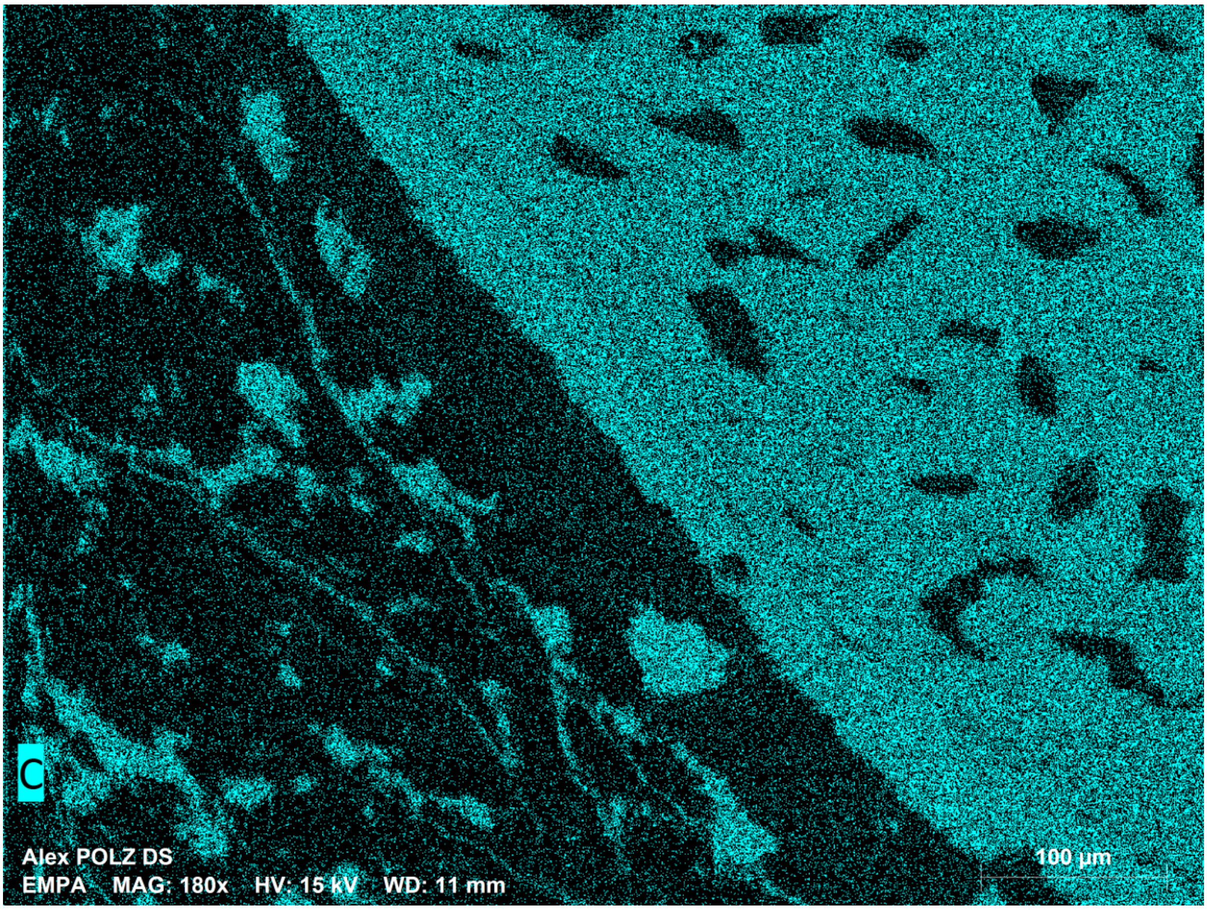

C
Alex POLZ DS
EMPA MAG: 180x HV: 15 kV WD: 11 mm
100 µm

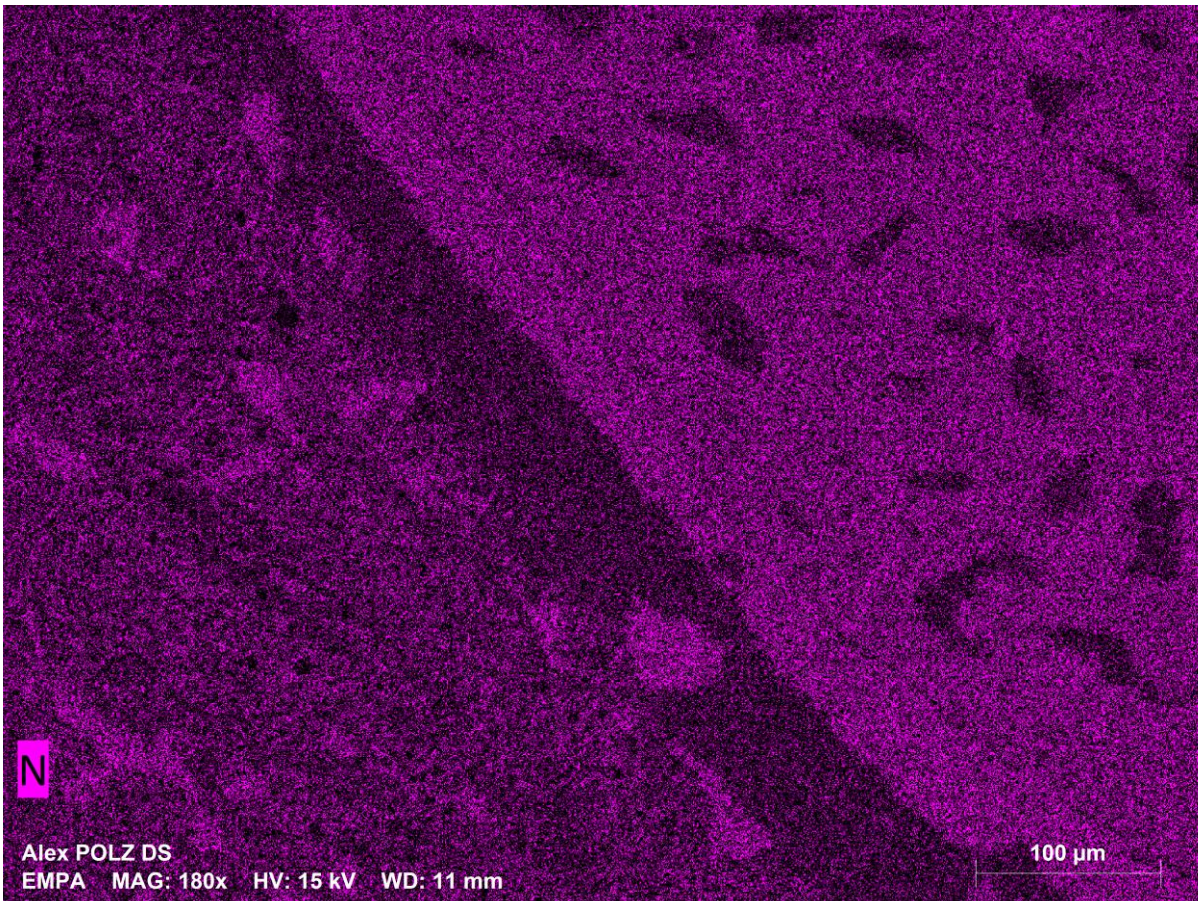
N
Alex POLZ DS
EMPA MAG: 180x HV: 15 kV WD: 11 mm
100 µm

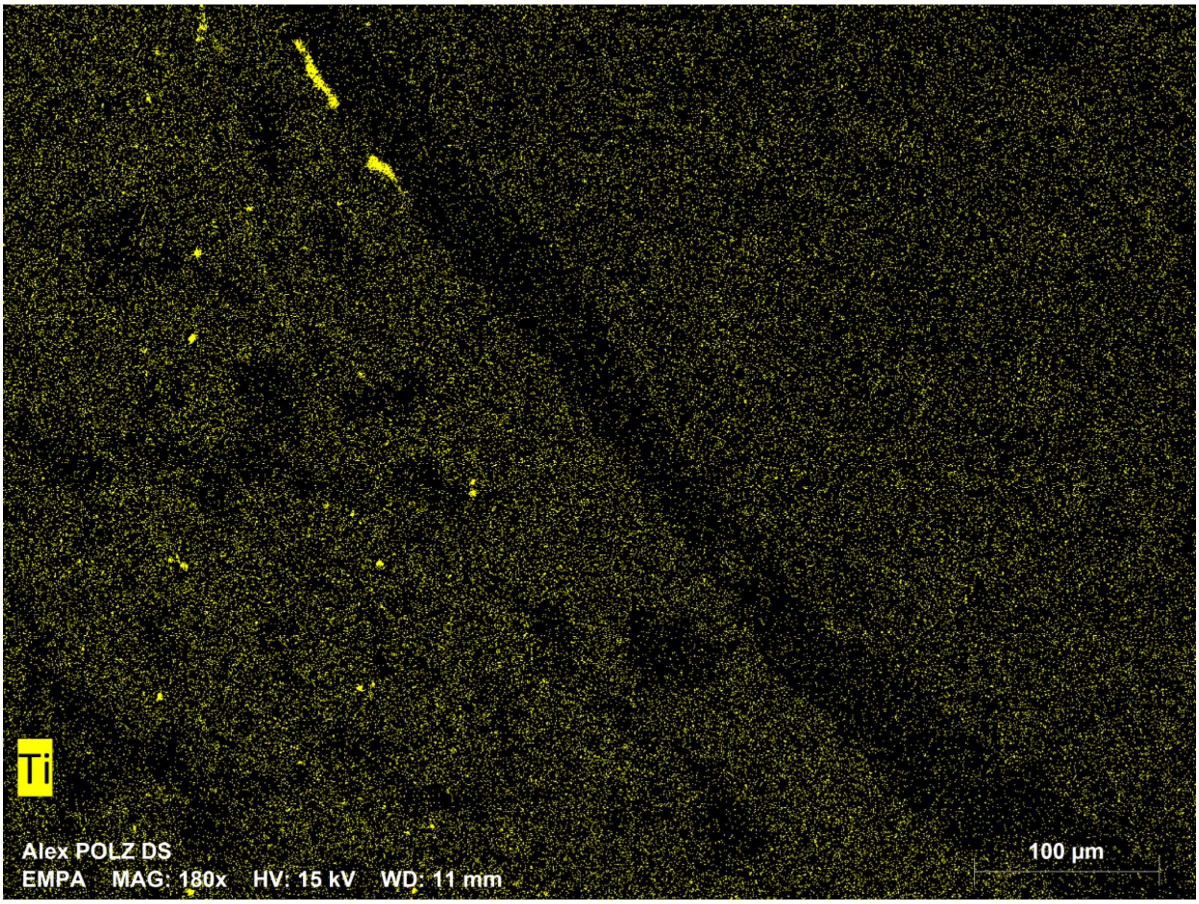
Ti
Alex POLZ DS
EMPA MAG: 180x HV: 15 kV WD: 11 mm
100 µm

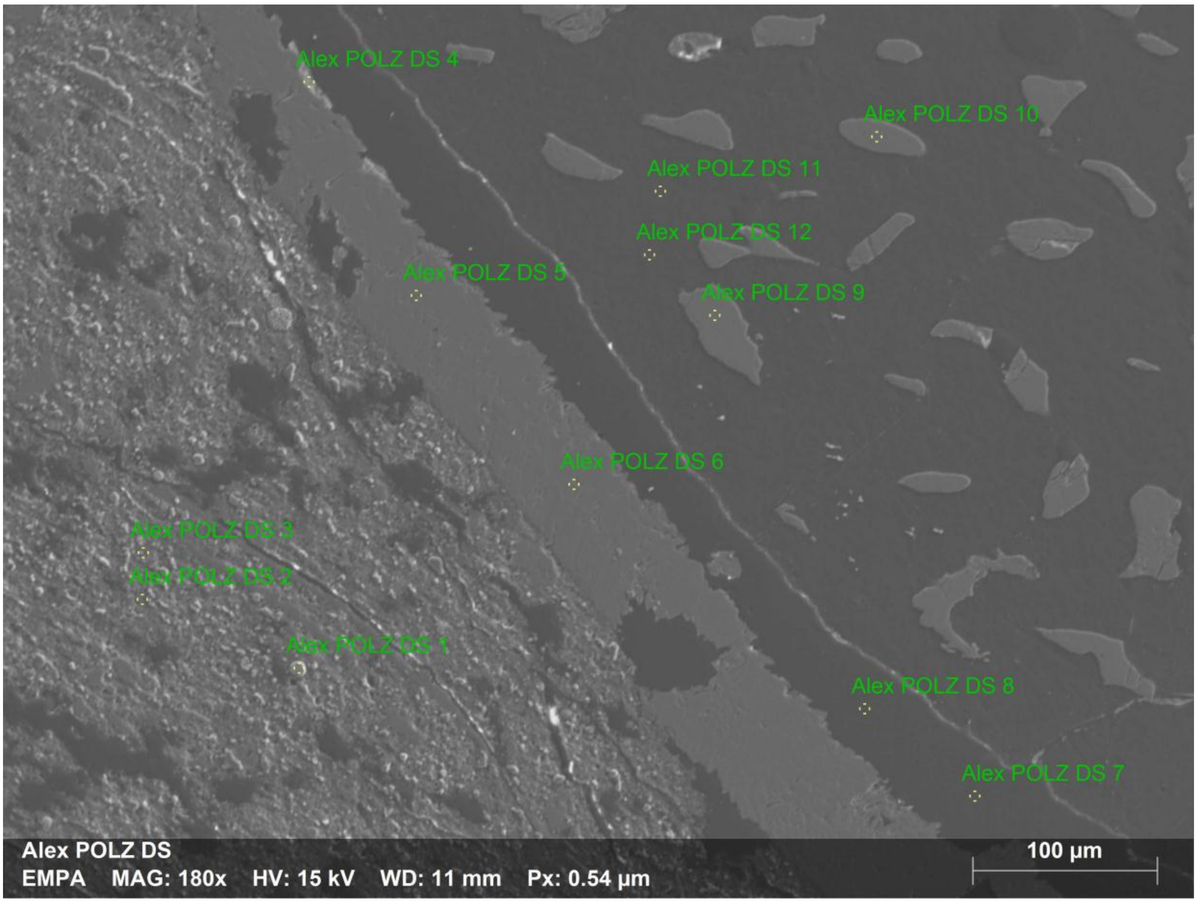
Alex POLZ DS 4
Alex POLZ DS 10
Alex POLZ DS 11
Alex POLZ DS 12
Alex POLZ DS 5
Alex POLZ DS 9
Alex POLZ DS 6
Alex POLZ DS 3
Alex POLZ DS 2
Alex POLZ DS 1
Alex POLZ DS 8
Alex POLZ DS 7
Alex POLZ DS
EMPA MAG: 180x HV: 15 kV WD: 11 mm Px: 0.54 µm
100 µm

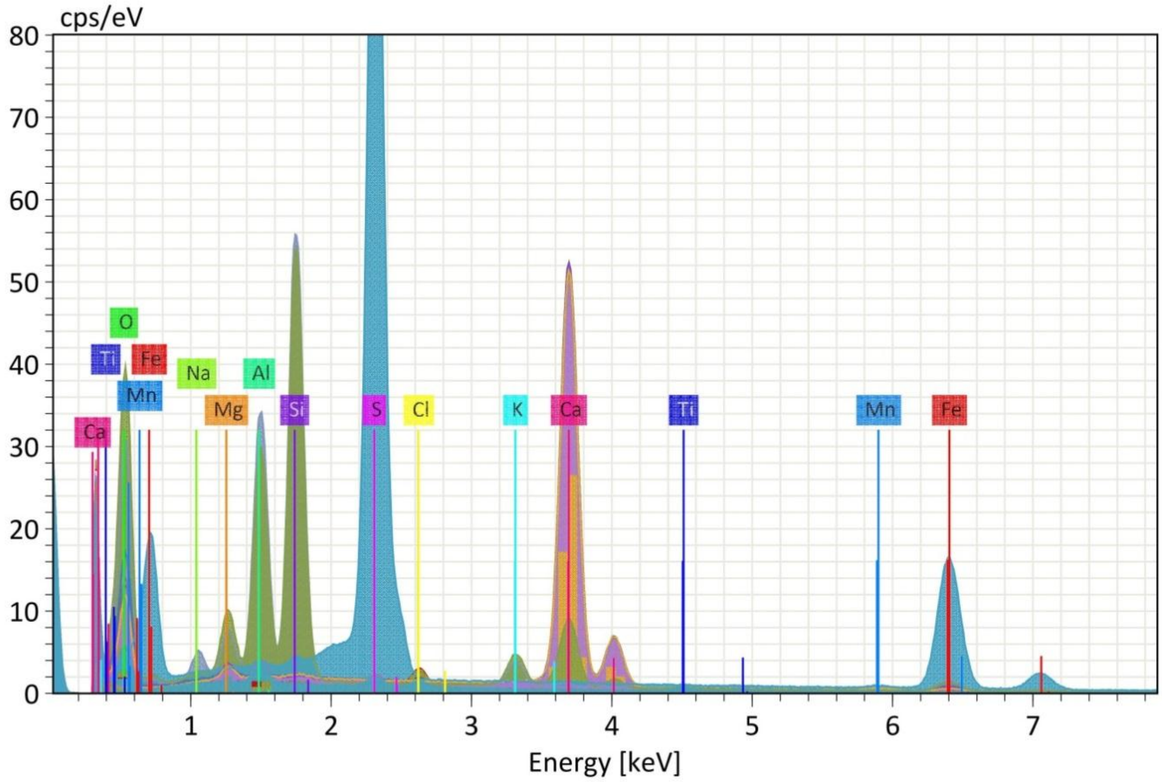
cps/eV
80
70
60
50
40
30
20
10
0
O
Ti
Fe
Mn
Ca
Na
Al
Mg
Si
S
Cl
K
Ca
Ti
Mn
Fe
1
2
3
4
5
6
7
Energy [keV]

Normalized stoichiometric concentration [%]

Spectrum	O	Cl	MgO	Al2O3	SiO2	CaO	Na2O	MnO	Fe2O3	TiO2	K2O	SO3
Alex POLZ DS 1	0.00		0.00	0.00	0.00	0.21	0.00	1.08	69.47	0.04	0.11	29.09
Alex POLZ DS 2	0.00		5.85	22.37	50.27	11.11	0.70	0.21	5.46	0.45	3.58	
Alex POLZ DS 3	0.00		4.16	26.36	56.00	0.59	2.63	0.11	6.81	0.16	3.17	
Alex POLZ DS 4	0.00		2.09	1.01	1.19	92.54	0.33	1.10	1.50	0.04	0.20	
Alex POLZ DS 5	0.00		2.05	0.98	0.91	93.55	0.53	0.44	1.28	0.02	0.25	
Alex POLZ DS 6	0.00		1.94	0.95	0.94	93.84	0.44	0.38	1.26	0.00	0.26	
Alex POLZ DS 7	0.00		11.20	58.97	17.56	2.11	8.96	0.00	0.00	0.96	0.25	
Alex POLZ DS 8	0.00	34.23	11.55	25.03	15.22	3.04	9.16	0.00	0.88	0.29	0.62	
Alex POLZ DS 9	0.00		2.35	0.86	0.89	91.25	0.27	0.78	3.34	0.08	0.18	
Alex POLZ DS 10	0.00		2.33	0.85	0.93	91.96	0.31	0.62	2.74	0.01	0.24	
Alex POLZ DS 11	0.00	7.04	6.79	34.46	5.39	2.08	6.17	0.00	1.01	11.08	0.00	25.98
Alex POLZ DS 12	0.00	6.95	7.30	32.76	6.27	2.21	7.07	0.07	1.13	11.39	0.19	24.67

Mass concentration [%]

Spectrum	O	Na	Mg	Al	Si	S	Cl	K	Ca	Ti	Mn	Fe	Sum
Alex POLZ DS 1	38.66	0.00	0.00	0.00	0.00	11.65		0.09	0.15	0.02	0.84	48.59	100.00
Alex POLZ DS 2	45.45	0.52	3.53	11.84	23.50			2.97	7.94	0.27	0.17	3.82	100.00
Alex POLZ DS 3	47.41	1.95	2.51	13.95	26.18			2.63	0.42	0.10	0.08	4.76	100.00
Alex POLZ DS 4	29.18	0.25	1.26	0.53	0.56			0.16	66.14	0.02	0.85	1.05	100.00
Alex POLZ DS 5	29.12	0.39	1.24	0.52	0.42			0.21	66.86	0.01	0.34	0.90	100.00
Alex POLZ DS 6	29.11	0.32	1.17	0.50	0.44			0.22	67.06	0.00	0.30	0.88	100.00
Alex POLZ DS 7	44.90	6.65	6.75	31.21	8.21			0.20	1.51	0.58	0.00	0.00	100.00
Alex POLZ DS 8	28.19	6.79	6.96	13.25	7.11		34.23	0.51	2.17	0.17	0.00	0.61	100.00
Alex POLZ DS 9	29.16	0.20	1.42	0.46	0.42			0.15	65.21	0.05	0.60	2.34	100.00
Alex POLZ DS 10	29.15	0.23	1.41	0.45	0.43			0.20	65.72	0.00	0.48	1.92	100.00
Alex POLZ DS 11	44.29	4.58	4.10	18.24	2.52			0.00	1.49	6.64	0.00	0.70	100.00
Alex POLZ DS 12	43.85	5.25	4.40	17.34	2.93			0.16	1.58	6.82	0.05	0.79	100.00
