# Supplementary material for: Mineralized belemnoid cephalic cartilage from the late Triassic Polzberg Konservat-Lagerstätte (Austria)
Source: PLoS One. 2022 Apr 20;17(4):e0264595. doi: 10.1371/journal.pone.0264595 (PMC9020720; doi:10.1371/journal.pone.0264595)
Supplement: Attachment — Submitted filename: Response to Reviewers.doc [file pone.0264595.s014.doc]

**Petra Lukeneder, M.Sc.**

University of Vienna

Althanstrasse 14A, A-1090 Vienna

e-mail: a0501032@unet.univie.ac.at

Vienna, 11 March 2022

**Reply Letter**

for the submitted article for publication in ***PLOS ONE***

**New title of submitted manuscript:** Mineralized belemnoid cephalic cartilage from the Late Triassic Polzberg *Konservat-Lagerstätte* (Austria)

**Dear editor of *PLOS ONE, dear reviewers,***

Thank you very much for your reviews!

Comments, annotations and suggested changes by the editor and 2 reviewers given directly in pdfs of the manuscript were ALL checked, and changes directly performed in the source word file. Following the suggestions of reviewer 2 suggestions we also had to change the title. We have enriched the data with most recent collected specimens (S1 table, S10 table).

Line numbers given below are those (old originals) from the annotated pdf before review, adaptation and corrections.

According to Christian Klugs (**Reviewer 1**) suggestions, we have added the chapter about homologization. Due to **Reviewer 2**’s input we came back to our original idea that Type B cartilages could be disarticulated parts of the arm cartilage (which is in fact, in modern coleoids, only loosely connected to other parts of the cartilage-complex). We have updated our reference list, due to changes in the order of the text, they are also ordered new.

**List of changes and annotations, comments and/or rebuttals to the reviewers comments on the submitted manuscript. Adaptations, changes, deleted parts and added parts are in the resubmitted manuscript with track change system. A clean, final version is also submitted.**

**Author’s revision process**, for a better confirmability of the review process.

**step 1**: adaptation and work on the annotations and comments of the editor

**step 2:** adaptation, reply and incorporation of the annotations and comments made by Reviewer 1 (Christian Klug)

**step 3:** adaptation, reply and incorporation of the annotations and comments made by Reviewer 2

**step 4:** transformation of the manuscript, checking and updating of the reference list, newer and better known references were added

**step 5:** composition of new and updated figures and completing supporting data (some new specimens added, data update)

**step 5:** composition of a reply letter

**step 6:** resubmission of the adapted manuscript

**Editors comments:**

1. ***Please ensure that your manuscript meets PLOS ONE's style requirements, including those for file naming. The PLOS ONE style templates can be found at*** [***https://journals.plos.org/plosone/s/file?id=wjVg/PLOSOne_formatting_sample_main_body.pdf***](https://deref-gmx.net/mail/client/X9RQVttdVls/dereferrer/?redirectUrl=https%3A%2F%2Fjournals.plos.org%2Fplosone%2Fs%2Ffile%3Fid%3DwjVg%2FPLOSOne_formatting_sample_main_body.pdf) ***and*** [***https://journals.plos.org/plosone/s/file?id=ba62/PLOSOne_formatting_sample_title_authors_affiliations.pdf***](https://deref-gmx.net/mail/client/Psng0f32Rw8/dereferrer/?redirectUrl=https%3A%2F%2Fjournals.plos.org%2Fplosone%2Fs%2Ffile%3Fid%3Dba62%2FPLOSOne_formatting_sample_title_authors_affiliations.pdf)

We have corrected the style requirements.

2. ***Please review your reference list to ensure that it is complete and correct. If you have cited papers that have been retracted, please include the rationale for doing so in the manuscript text, or remove these references and replace them with relevant current references. Any changes to the reference list should be mentioned in the rebuttal letter that accompanies your revised manuscript.***

Thank you, we have reviewed and corrected our reference list. We have also included further references, as suggested by the reviewers, in our list.

3. **In your manuscript, please provide additional information regarding the specimens used in your study. Ensure that you have reported specimen numbers and complete repository information, including museum name and geographic location.**

**If permits were required, please ensure that you have provided details for all permits that were obtained, including the full name of the issuing authority, and add the following statement: 'All necessary permits were obtained for the described study, which complied with all relevant regulations.' If no permits were required, please include the following statement: 'No permits were required for the described study, which complied with all relevant regulations.'**

We have added the corresponding statement to the Material and methods section.

**4.	We note that the grant information you provided in the ‘Funding Information’ and ‘Financial Disclosure’ sections do not match. When you resubmit, please ensure that you provide the correct grant numbers for the awards you received for your study in the ‘Funding Information’ section.**

We have corrected the funding information!

4. **We note that Figure 1 and SI Fig 1 in your submission contain [map/satellite] images which may be copyrighted. All PLOS content is published under the Creative Commons Attribution License (CC BY 4.0), which means that the manuscript, images, and Supporting Information files will be freely available online, and any third party is permitted to access, download, copy, distribute, and use these materials in any way, even commercially, with proper attribution. For these reasons, we cannot publish previously copyrighted maps or satellite images created using proprietary data, such as Google software (Google Maps, Street View, and Earth). For more information, see our copyright guidelines:** [**http://journals.plos.org/plosone/s/licenses-and-copyright**](https://deref-gmx.net/mail/client/ws7UCfaObDo/dereferrer/?redirectUrl=http%3A%2F%2Fjournals.plos.org%2Fplosone%2Fs%2Flicenses-and-copyright)**.**

We have removed these figures from the submission and prepared new ones!

**Reviewer 1:** Christian Klug

***Line 1: I usually write cephalic cartilage...***

We have now adopted this term.

***Line 29: a bit trivial***

We have adapted the text.

***Line 32: This comes a bit out of the blue. You need a sentence to provide a trnaistion from your studies to morphological details.***

We have adapted the text.

***Line 35: From my humble point of view, to fail to mention the most interesting result of your study: The homologisation of the fossil parts with parts of the cephalic cartilage of modern coleoids. I would add a sentence mentioning this.***

We have corrected the text.

***Line 47: The cephalopod cephalic cartilage was also described by Fuchs from Lebaneses coleoids, by myself from Carboniferous coleoids and ammonoids from the Teutoburger Wald and Eichstätt. I just saw that you expand on this below. Maybe mention this here.***

We have now switched much about the fossil record to this section.

***Line 87: What does this mean? In which respect? Functionally incomplete? Not closed?***

It is not closed, we have corrected the sentence.

***Line 165: Are these generic names up-to-date in these combinations? If not, use '...'!***

We have added inverted commas.

***Line 287: I don't understand what you mean by sear here. Patch? Halo?***

Seam, a “m” was missing, we have corrected this.

**Line 307: six Type A fossils are associated with hooks, six others with a coleoid proostracum.**

Yes, there is historical material known from Cave del Predil, described by Mojsisovics and Suess, where the cartilages are in situ preserved with coleoid hooks and proostraca. There are also specimens in collections of the NHMW where there are all three aspects preserved. We have added this information and an additional Supporting Table S2.

***Line 310: Are the proportions proostracum length/ distance similar/ identical or do they vary strongly?***

We have added this information in an additional Supporting Table S2.

**Line 316: The C structure to processus ratio ranges from 0.7 to nearly 1.4.**

This aspect is now also discussed in the chapter about homologization.

***Line 475: this is a conclusion, which I would put rather at the end of that chapter.***

We have adapted this and moved it to the end of the chapter.

***Line 488: This chapter is very short but very important, I feel. I would provide a bit more detail homologising details of the cartilages in yours and living coleoids.***

We renamed the chapter (change to: Homologisation with the anatomy of modern coleoids) and provided more information.

***Line 518: this needs to be elaborated a bit more in the main text!***

We have rephrased this sentence.

**Reviewer 2:** anonymous reviewer

***Line 39: a short introduction into the role of phragmoteuthids in the evolution of crown coleoids would be very informative***

Adapted, you can find a short section about this from line now from line 58 to line 62.

***Line 57: would discuss this in the end......***

Adapted, we have now changed to a more catchy sequence from fossil to recent. Now we have a very nice transition from Phragmoteuthids to the Polzberg *Konservat-Lagerstätte*

***Line 69: I recommend to add better known, more recent references to support this phylogenetic relationship.***

We have adapted this and use newer information about systematic relationships.

***Line 81: the animals you are referring include more than only squids, which is a common name for only teuthids***

We have corrected this mistake.

***Line 113: please note that remains of fin & nuchal cartilages are also described***

We have adapted this!

***Line 129: reference for Boreopeltis, Rhomboteuthis, Vampyronassa, Proteroctopus, Mastigophora?***

***these taxa are not listed in Donovan & Fuchs***

We have corrected this mistake!

***Line 143: I´m confused....."Reingraben" or "Raingraben" (see Doguzhaeva?) "Rinngraben", Cave de Predil (see supplementary) is not "Reingraben"?***

Reingraben shales refer to the Raingraben type locality at Rohr im Gebirge (Lower Austria); anyway the lithostratigraphic unit was named “Reingraben shales” and not “Raingraben shales” Unfortunately there are no coleoid specimens reported from Rohr im Gebirge. Unfortunately not all authors follow these recommendations from the Stratigraphic chart of Austria and so we also find designations like “Raingraben shales” or “Reingrabner Schiefer”.

In contrast, Rinngraben, also called “Rinnengraben”, is a locality near Cave del Predil, from which Suess has described Phragmoteutids with the here studied black structures. The german name originates from the 19^th^ century when it was part of Carinthia (Cave del Predil was called Raibl then).

***Line 152: what mark the red stars?***

We have changed Figure 2 and added a description for the red stars.

***Line 172: how old? where in your stratigraphic chart?***

We have added the age of deposits in Cave del Predil.

***Line 184: Apparently, there exists confusion about material from the locality "Lunz"....***

***so, your specimens are exclusively from the Reingraben Shales of Lunz (& Cave de Predil); no specimens from the overlying Lunz Formation of "Lunz", right? Did you find cranial cartilages in specimens from the Lunz Formation? Where are the specimens of Doguzhaeva et al. (2007) from? Reingraben Shales or Lunz Formation?***

Historical specimens from Polzberg were often blurrly described as “Lunz” (19^th^ century), because the localities and units were not subdivided then (it was all “Lunz”). All our specimens come from Polzberg (historical and new finds from 2021) and Raibl (historical material).

The specimens from Doguzhaeva et al. (2007) seem to come from basal layers of Polzberg (but again: she has only used historical material).

Interestingly Doguzhaeva & Summesberger (2012) has published a *Phragmoteuthis bisinuata* from Schindelberg (= Polzberg –it is exactly the same locality but it is known under both names) - NHMW 2006z0235/0054 - a number which does not exist in the NHMW database- and: which is from Raibl (Cave del Predil). Exactly the same specimen (negative) is figured in Suess Tafel 1 Fig. 2a and Mojsisovics Taf. XCIV Fig. 3. The lithology from deposits in Raibl and Polzberg is slightly different, but there is also a label with the locality present, so we do not know why she has mixed them up. The specimen in Doguzhaeva & Summesberger (2012), p. 33 Fig. 1 is definitely from Raibl, and not from Schindelberggraben/Polzberg. As the *Lunzoteuthis* in Fig. 2 was sputtered, we cannot say much about the origin, lithology on the backside of SEM specimens looks like deposits at Polzberg. What we know is that she has it from historical material at the NHMW.

***Line 308: any description possible?***

We have added a description.

***Line 310: belemnoids have a proostracum, octopods and squids a gladius***

Of course, we have corrected this mistake.

***Line 312: are they figured? if not, I recommend to show them the reader; if yes, where in Fig 3?***

Yes, they are figured in Fig. 3C, we have added a link.

**Line 314: I guess you mean the diameter of the C structure?**

We have concretized.

**Line 321:**

We have corrected this description.

***Line 323: where in figure 3C-D***

We have added the reference to Fig. 2A.

***Line 334: catalog no.?***

We have added the inventory number.

***Line 344: I do not agree, because this complex structure is unlike what we know about belemnoid mega hooks.......apart from this: mega hooks are so far unknown from Phragmoteuthida!***

Due to your input, we came back to our original idea, that these are the disarticulated arm cartilage elements.

***Line 352: here in this results section you avoid to name the studied structures "cartilage"; I understand this practice, but you do in the capture of figure 5, which I find is inconsistent***

We have corrected this!

***Line 393: I recommend to declare here that you interpret these carbonised structures to represent cranial cartilages.....or.....you already state in the results section that you are dealing with the cranial cartilage (which is actually already done in the introduction...)***

We decided to keep the original order, because we think the ideas are probably easier to follow. The topic of invertebrate cartilage seems to be “exotic” for many researchers and we hope, also to attract a broad spectrum of readers.

**Line 393: I would refer to the chemical composition after you have discussed Type 1+2.**

We have added a corresponding section.

**Line 398: to support this idea, I would figure at least the microhooks & proostracum Is the proostracum 3-parted, which is typical for phragmoteuthid belemnoids.**

We have now figured belemnoid specimen with hooks in Figure 2.

**Line 426: ? where in figure?**

We have corrected this sentence.

***Line 432: a figure would be nice.....***

We have added a Supporting figure S (*Loligo vulgaris* specimen and recent cephalic cartilage).

**Line 436: please provide reference**

Own observation during dissections – we have added a corresponding note.

**Line 438: I disagree with this interpretation, because in my experience these structures have nothing in common with belemnoid mega hooks...What are the unlabeled curved structures in figures 3C-D & 4C-D, Fig. 4C e.g. is in my eyes Type A**

We took these ideas into account and reconsidered this part of the manuscript. Regarding the curvature of Type A fossils all show a very constituent shape and course, which differs strongly from Type B. We came back to our original idea, that these fossils are disintegrated elements of the arm cartilage.

**Line 455: Are mega hooks known to bear a channel system?**

No not at all, but a cartilaginous structure of megahooks was discussed; this probably would include a channel system.

***Line 494: what is the label cs?***

This is the calcite seam, a corresponding lettering can be found in the caption.

***Line 513: the 1st 3d (unflattened), 1st carbonized, but 1st detailed? in specimens from plattenkalks you can recognize the oesophagal foramen in the cartilage.....what is the more "detailed report"? I think one cannot compare different kind of preservations.....***

We have corrected this sentence.

***Line 423: located close to proximal arm hooks?***

We have corrected this mistake.

***Line 531: not sure what you mean by "enigma"?***

We have concretized this sentence.

***Line 452: as said I do not agree, but if you are sure with your idea of mega hooks, you can state that this is the first record of mega hooks with phragmoteuthid belemnoids.....***

We returned to our original idea, which is also for us more consistent.

Dear academic editor, dear Steffen Kiel

We adapted all points raised by the editor, reviewer 1 (Christian Klug) and reviewer 2 (anonymous). We appreciate the fair and accurate reviews and comments and it is certain that the quality of the submitted paper was raised by the adaptations. We hope that you are also satisfied with our adaptations and work on the manuscript and hope that you will come to a positive decision for a publication in PLOS ONE.

Yours sincerely, the authors

Petra Lukeneder & Alexander Lukeneder
