# Supplementary material for: Mineralized belemnoid cephalic cartilage from the late Triassic Polzberg Konservat-Lagerstätte (Austria)
Source: PLoS One. 2022 Apr 20;17(4):e0264595. doi: 10.1371/journal.pone.0264595 (PMC9020720; doi:10.1371/journal.pone.0264595)
Supplement: Attachment — Submitted filename: Response to Reviewers.doc [file pone.0264595.s016.doc]

**Petra Lukeneder, M.Sc.**

University of Vienna

Althanstrasse 14A, A-1090 Vienna

e-mail: a0501032@unet.univie.ac.at

Vienna, 15 March 2022

**Reply Letter**

for the submitted article for publication in ***PLOS ONE***

**Title of submitted manuscript:** Mineralized belemnoid cephalic cartilage from the Late Triassic Polzberg *Konservat-Lagerstätte* (Austria)

**Dear editor of *PLOS ONE, dear Steffen Kiel,***

Thank you very much for your review! Your comments annotations and suggested changes given directly in the manuscript were all checked, and changes directly performed in the source word file. According to your suggestions, we have also changed the figures 3, 7 and 8. All figures have been unified. References have been checked.

Line numbers given below are those (old originals) from the annotated word file before review, adaptation and corrections.

**List of changes and annotations, comments and/or rebuttals to the edtors comments on the submitted manuscript. Adaptations, changes, deleted parts and added parts are in the resubmitted manuscript with track change system. A clean, final version is also submitted (File: Manuscript).**

**Author’s revision process**, for a better confirmability of the review process.

**step 1**: adaptation and work on the annotations and comments of the editor

**step 2:** transformation of the manuscript

**step 3:** composition of updated figures

**step 4:** composition of a reply letter and updating cover letter

**step 5:** resubmission of the adapted manuscript

**Editor comments:**

***Line 76: [why again]***

Here we wanted to point to the different designations of Polzberg locality. In historical collections and literature it is also known as “Schindelberg”, “Schindlberg” (different spelling) and Lunz. But we have now deleted this part! It might be more confusing.

***Line 78 and line 80: ..over…***

We have adapted the text!

***Line 86: [not sure what’s meant by this – perhaps remove?]***

We have deleted this part!

***Line 114:***

We have deleted the repetitive part!

***Line 180:***

We have concretized the sentence!

***Line 240:***

We have adapted the chapter title.

***Line 349:***

We have adapted the sentence for a better understanding.

***Line 382:***

We have changed Figure 3.

***Line 367:***

We have changed the expression.

***Line 401:***

We have adapted the sentence for a better understanding.

***Line 408:***

We have changed the expression.

***Line 425:***

We have adapted Figure 7.

***Line 552:***

We have adapted the expression.

***Line 553:***

We have adapted the expression.

***Line 571:***

We have adapted Figure 8.

***Line 687:***

As “bromalites” is not a genus name, we have not adapted this suggestion.

***Line 710:***

We have corrected the references according to the editors suggestions.

***Line 718:***

We have corrected the references according to the editors suggestions.

***Line 741:***

We have corrected the references according to the editors suggestions.

***Line 752:***

We have deleted the duplicated part.

***Line 763:***

We have corrected the references according to the editors suggestions.

Dear academic editor, dear Steffen Kiel

We corrected almost all points (except line 687) raised by the editor Steffen Kiel. We are very thankful for the fair and accurate comments and we feel certain that the quality of the manuscript was raised by the adaptations! We hope that you are satisfied with our work on the manuscript and hope that you will come to a positive decision for a publication in PLOS ONE.

Yours sincerely, the authors

Petra Lukeneder & Alexander Lukeneder
